# Mining MYB transcription factors from the genomes of orchids (*Phalaenopsis* and *Dendrobium*) and characterization of an orchid R2R3-MYB gene involved in water-soluble polysaccharide biosynthesis

**DOI:** 10.1038/s41598-019-49812-8

**Published:** 2019-09-25

**Authors:** Chunmei He, Jaime A. Teixeira da Silva, Haobin Wang, Can Si, Mingze Zhang, Xiaoming Zhang, Mingzhi Li, Jianwen Tan, Jun Duan

**Affiliations:** 1 Key Laboratory of South China Agricultural Plant Molecular Analysis and Gene Improvement, South China Botanical Garden, Chinese Academy of Sciences, Guangzhou, 510650 China; 2 P. O. Box 7, Miki-cho post office, Ikenobe 3011-2, Miki-cho, Kita-gun, Kagawa-ken 761-0799 Japan; 3 University of the Chinese Academy of Sciences, Beijing, 100049 China; 4 Biodata Biotechnology Co. Ltd, Heifei, 230031 China; 5 College of Forestry and Landscape Architecture, South China Agricultural University, Guangzhou, 510642 China

**Keywords:** Gene expression, Secondary metabolism

## Abstract

Members of the MYB superfamily act as regulators in a wide range of biological processes in plants. Despite this, the MYB superfamily from the Orchidaceae has not been identified, and MYB genes related to bioactive water-soluble polysaccharide (WSP) biosynthesis are relatively unknown. In this study, we identified 159 and 165 MYB genes from two orchids, *Phalaenopsis equestris* and *Dendrobium officinale*, respectively. The MYB proteins were classified into four MYB classes in both orchids: MYB-related (MYBR), R2R3-MYB, 3R-MYB and atypical MYB proteins. The MYBR proteins in both orchids were classified into five subfamilies and 12 genes were strongly up-regulated in response to cold stress in *D*. *officinale*. The R2R3-MYB proteins were both divided into 31 clades in *P*. *equestris* and *D*. *officinale*. Among these clades, nine contained MYB TFs related to secondary cell wall biosynthesis or testa mucilage biosynthesis in *Arabidopsis thaliana*. In *D*. *officinale*, 10 candidate genes showed an expression pattern corresponding to changes in the WSP content. Overexpression of one of these candidate genes (*DoMYB75*) in *A*. *thaliana* increased seed WSP content by about 14%. This study provides information about MYB genes in two orchids that will further help to understand the transcriptional regulation of WSP biosynthesis in these orchids as well as other plant species.

## Introduction

Gene expression is regulated by various complex mechanisms, including modifications to DNA such as histone modification and DNA methylation, as well as various RNA-mediated processes. Transcription factors (TFs) regulate gene expression, and this is a well-known mechanism by which a TF binds to a specific nucleotide sequence upstream of target gene, ultimately controlling a range of biological processes^1^. MYB TFs exist widely in eukaryotes and are one of the largest and most diverse families of TFs in the plant kingdom, where they play an essential role in a wide range of physiological and biochemical processes^2,3^.

The first MYB gene (*v*-*myb*), which was isolated from avian myeloblastosis virus (AMV), encodes a MYB domain protein^4^. Ever since the first plant MYB gene (the *Zea mays COLORED1* (*C1*) gene) was cloned^5^, numerous MYB genes have been identified from plants as an increasing number of plant genomic sequences became available. For example, 198, 256, 127, 231 and 122 MYB genes have been identified in *Arabidopsis thaliana*^6^, *Brassica rapa*^3^, *Solanum lycopersicum*^7^, *Pyrus bretschneideri*^8^ and *Brachypodium distachyon*^9^, respectively. MYB proteins share a highly conserved DNA-binding domain (the MYB domain), which ranges from one to four imperfect amino acid sequence repeats (R)^10^. Based on the number of adjacent repeats, the MYB genes in plants have been divided into four distinct groups: MYB-related (MYBR, only contains one R1- or R2-like repeat), R2R3-MYB (containing two R2/3R-like repeats), 3R-MYB (containing three R1/R2/R3-like repeats) and atypical MYB proteins (4R-MYB, four R1/R2-like repeats; CDC5-like)^6,10^. The atypical MYB group of proteins is the smallest class, and contains one or two genes in several higher plant genomes. For example, one and two atypical MYB genes were found in *O*. *sativa* and *A*. *thaliana*, respectively^11^. The 3R-MYB group is the second smallest class, and contains about five members in plants such as *A*. *thaliana*, *Populus trichocarpa* and *Vitis vinifera*^3^. The MYBR proteins contain only a single repeat and fall into five subclasses, CCA1/R-R-like, CPC-like, I-box-like, TBP-like and TRF-like, that contain 30 to 70 genes in plant genomes^2^. The R2R3-MYB group is the largest group in plants with more than 100 members that were extensively amplified approximately 500 million years ago after the appearance of land plants^12^. For example, 113, 126 and 244 genes encode R2R3-MYB proteins in *O*. *sativa*^11^, *A*. *thaliana*^6^ and *Glycine max*^13^, respectively. The *A*. *thaliana* R2R3-MYB proteins have been divided into 33 clades, while those in *P*. *bretschneideri* have been divided into 37 clades^8^, which suggests evolutionary diversity of the R2R3-MYB family.

All four groups of MYB proteins were found after genome-wide analyses of MYB TFs in plants and while the function of a number of MYB genes have been characterized in many plants, the function of 4R-MYB proteins remains unclear. The 3R-MYBs play a role in cell cycle control^14^. MYBR proteins are involved in cellular morphogenesis, secondary metabolism, organ morphogenesis, phosphate starvation, chloroplast development and circadian regulation^15^. More recently, two *A*. *thaliana* MYBR genes (*MYBS1* and *MYBS2*) have been shown to have opposite roles in sugar signaling mechanisms^16^. Over the past two decades, the R2R3-MYB TFs have been extensively exploited and many R2R3-MYB proteins have been shown to play roles in several biological processes, such as development, response to biotic and abiotic stresses, and metabolism^15,17^. Among the metabolic processes, R2R3-MYB genes are acutely involved in phenylpropanoid metabolism^18^. Very recently, two R2R3-MYB genes from *Marchantia polymorpha*, *MpMYB14* and *MpMYB02*, were found to act as essential regulators in the biosynthesis of riccionidins and marchantins, respectively^19^.

Secondary cell walls (SCWs), which have a critically important function by supporting plants and are a major source of plant biomass, are composed of cellulose, lignin and hemicellulose^20^. An increasing body of studies has demonstrated that R2R3-MYB proteins are critical for SCW biosynthesis^21^. In *A*. *thaliana*, AtMYB46 binds to the promoter of *AtCSLA9*, which is involved in glucomannan biosynthesis, and regulates its expression^22^. Testa mucilage, which is composed of polysaccharides, is regarded as a useful model for exploring the biosynthesis of cell wall polysaccharides^23,24^. Previous studies have demonstrated that R2R3-MYB genes, such as *AtMYB5* and *AtMYB61*, are required for the production of seed mucilage, and the polysaccharide content of seed mucilage in the *A*. *thaliana myb61* mutant was significantly reduced^25,26^. These results indicate that R2R3-MYB members play roles in the biosynthesis of plant polysaccharides.

Water-soluble polysaccharides (WSPs) play an important role in plants’ stress response. For example, tolerant genotypes of wheat (*Triticum aestivum* L.) seedlings accumulated more water-soluble carbohydrates, including glucose, fructose, sucrose, and fructan than sensitive genotypes under drought and salt stress^27^. Recent studies showed that WSPs isolated from plants may increase immunity^28^ and have an antitumor function^29,30^. The Orchidaceae is one of the largest plant families in the world and has about 25,000 species^31^. The *Dendrobium* genus, which belongs to the Orchidaceae, has several important species that are used as herbal medicines^32^. WSPs isolated from *Dendrobium* species such as *D*. *huoshanense* and *D. officinale* are regarded as major active ingredients and display immunomodulating activities^33^. Several genes involved in the biosynthesis of WSPs have been identified and characterized in *D*. *officinale*, whose stems contain an abundance of bioactive WSPs^34–36^. However, the TFs that regulate the biosynthesis of these WSPs are still unknown. In this study, MYB proteins were identified from two orchids, *Phalaenopsis equestris* and *D*. *officinale*, in a genome-wide process, and the putative R2R3-MYB genes related to SCW or mucilage biosynthesis were identified based on phylogenetic analysis. One R2R3-MYB gene involved in the biosynthesis of WSPs was characterized. This work provides novel information that would allow for a better understanding of the functional diversity of MYB genes in plants and would aid in revealing the molecular mechanisms underlying the biosynthesis of bioactive WSPs in *D*. *officinale* or in other plants.

## Methods

### Plant materials and treatments

*D. officinale* plants were grown as described previously^34^. The stems of five developmental stages were harvested to determine WSPs and for gene expression analysis. S1 is about 4 months after sprouting in April, while S2, S3, S4, and S5 are about 9, 10, 12, and 13 months after sprouting, respectively. Roots, leaves and stems that were collected from plants grown in a growth chamber when they were 10 cm in height, were used to analyze the expression pattern of different organs. *D*. *officinale* seed capsules were surface sterilized in 0.1% mercuric chloride (HgCl_2_), sown on half-strength Murashige and Skoog medium (half the macronutrients; ½MS)^37^ supplemented with 1 g/L activated charcoal, 20 g/L sucrose and 6 g/L agar and 0.5 mg/L 1-naphthalene-acetic acid (NAA), and cultivated at 26 ± 1 °C, 40 µmol m^−2^ s^−1^, and a 12-h photoperiod. Seedlings (1 cm in height and about 3 months after sowing) were transferred to liquid ½MS medium supplemented with 20 g/L sucrose and 0.5 mg/L NAA for three days. After adapting, 30 seedlings were used to perform abiotic stress bioassays in liquid medium containing 150 g/L polyethylene glycol (PEG) 6000 (Sigma-Aldrich, Shanghai, China), 300 mM mannitol (Sigma-Aldrich), or 250 mM NaCl (Guangzhou Chemical Reagent Factory, Guangzhou, China). Seedlings were transferred to fresh ½MS medium supplemented with 20 g/L sucrose and 0.5 mg/L NAA as the control. There were three replicates with 36 seedlings in each treatment. After 6 h, seedlings were harvested, frozen in liquid nitrogen and RNA was isolated immediately.

*A. thaliana* plants (Col-0) were grown in soil under a 16-h photoperiod at 22 °C. For screening resistant lines, *A. thaliana* seeds were surface sterilized and sown on ½MS medium supplemented with 15 g/L sucrose and 8 g/L agar, stratified in the dark at 4 °C for 2 d, and then cultivated under a 16-h photoperiod at 22 °C.

### Identification of MYB transcription factors in orchids

The protein sequences of *P*. *equestris* and *D*. *officinale* were downloaded in a FASTA format from orchidbase (http://orchidbase.itps.ncku.edu.tw/EST/releaseSummary2012.aspx) and the National Center for Biotechnology Information (NCBI) provided by Zhang *et al*.^38^. HMMER 3.0 software (http://hmmer.janelia.org/) was used to identify the putative MYB TFs under default parameters. The putative MYB TFs were annotated by Pfam (Protein family)^39^, Swissprot^40^ and nr (NCBI non-redundant protein sequences)^41^. The putative MYB TFs, which were confirmed to be MYB TFs by annotation, were regarded as MYB TFs. The classification of MYBR, R2R3-MYB, 3R-MYB and atypical MYB proteins were based on the annotation and BLAST against the *A. thaliana* MYB TFs.

### Phylogenetic analysis

MYB proteins from *A*. *thaliana* (At), *P. equestris* (Pe) and *D*. *officinale* (Do) were aligned using MAFFT software version 7^42^. A phylogenetic tree of R2R3-MYBs was constructed using the Neighbor–Joining (NJ) method and 1,000 bootstraps with Clustalx^43^. The phylogenetic trees of MYBR and C2 (S6) R2R3-MYBs were constructed using MEGA 7^44^ with the NJ method using 1,000 bootstraps.

### Calculation of Ks and Ka of MYB family genes in the two orchids

Orthologous gene pairs of MYB genes between *P*. *equestris* and *D*. *officinale* were identified by Orthofinder v2.2.6 with the BLAST method under default parameters^45^. The orthologous gene pairs were used to calculate synonymous (Ks) and nonsynonymous (Ka) values using the KaKs_Calculator2.0^46^.

### Prediction of *cis*-responsive elements on the promoters of orchid MYB genes

The 2000 bp genomic DNA sequences upstream of the initiation codon (ATG) of orchid MYB genes were obtained and used for predicting *cis*-acting regulatory DNA elements (*cis*-responsive elements). The PlantCARE database^47^ and PLACE database^48^ were adopted to identify the putative *cis*-responsive elements.

### Expression profiling of MYB genes from *D*. *officinale* under cold stress

For the expression profiles of *D*. *officinale* under cold stress (4 °C), the transcriptome sequencing data of the control condition (SRR3210630, SRR3210635 and SRR3210636) and cold stress treatment (SRR3210613, SRR3210621 and SRR3210626) were obtained from the NCBI Sequence Read Archive (SRA) database^49^. The clean reads were obtained by filtering out low quality reads and were mapped to the nucleotide sequences of MYB genes using TopHat version 2.0.8^50^. The expression level of MYB genes was calculated by the fragments per kilobase of exon per million fragments mapped (FPKM) method using HTSeq^51^. The heatmap of expression profiling was draw by a green-red gradient in R version 3.4.1 (https://www.r-project.org/). The genes with a FPKM value >5 in the control or cold stress treatment were regarded as sense, then were used to calculate fold change (mean of FPKM cold/mean of FPKM control). Genes with a ≥1.5-fold change were defined as up-regulated genes, and those with a ≤0.66-fold change were regarded as down-regulated genes.

### Quantitative RT-PCR (qRT-PCR) analysis

Total RNA from *D*. *officinale* organs (roots, stems and leaves) and seedlings, as well as *A*. *thaliana* seedlings, was extracted using an RNA extraction kit (Column Plant RNAout2.0, Tiandz, Inc., Beijing, China). RNA was purified by excluding genomic DNA using the DNase I digestion kit (Takara Bio Inc., Dalian, China). The integrity and content of purified RNA was determined by 1% agarose gel electrophoresis and a NanoDrop 2000c Spectrophotometer (Thermo Scientific, Wilmington, NC, USA), respectively. Total RNA was reversed transcribed into cDNA by M-MLV reverse transcriptase (Promega, Madison, WI, USA) according to the manufacturer’s protocol. The cDNA of each sample was diluted to 200 ng/mL and 1 μL was used as template for the qRT-PCR reaction. Three PCR reactions were performed using the SoAdvanced™ Universal SYBR^®^ Green Supermix detection system (Bio-Rad, Hercules, CA, USA) in an ABI 7500 Real-time system (ABI, Foster City, CA, USA) with the following amplification regime: 95 °C for 2 min, and 40 cycles of 95 °C for 15 s and 60 °C for 30 s. Actin from *D*. *officinale* (NCBI accession number: JX294908) was used to normalize the expression of genes. The 2^−ΔΔCT^ method^52^ was used to calculate the relative gene expression level. All the primers of *DoMYB* genes and actins for qRT-PCR were designed by an online web tool (http://www.idtdna.com/Primerquest/Home/Index) and are listed in Supplementary Table 1.

### Generation of *DoMYB75* transgenic lines

The coding sequence (CDS) of *DoMYB75* without a termination codon was amplified using the KOD FX High Success-rate DNA polymerase Kit (Toyobo Biotechnology Co. Ltd., Shanghai, China) and cloned into the pCAMBIA 1302 vector (Cambia, Canberra, Australia) at the *Nco*І site. The construct was verified by DNA sequencing at the Beijing Genomics Institute (Shenzhen, China). *A*. *thaliana* was transformed by the floral dip method^53^ using about 15 independent plants. Twenty five resistant lines were identified by screening in ½MS medium supplemented with 25 mg/L hygromycin B (Roche Diagnostics, Mannheim, Germany). Three resistant lines were randomly selected to extract genomic DNA and verified as transgenic lines using PCR. The 2 × BlueStar™ PCR Master Mix kit (Tingke Biotechnology Co. Ltd., Beijing, China) was used to perform PCR with 95 °C for 2 min and 35 cycles of 98 °C for 10 s, 58 °C for 30 s, and 72 °C for 30 s, followed by a final extension at 72 °C for 10 min. The primers used to construct the overexpression vector are listed in Supplementary Table 1.

### Analysis of *DoMYB75* transcript level in wild type and *DoMYB75* transgenic *A*. *thaliana* plants

Total RNA from one-week-old *A*. *thaliana* seedlings were extracted, purified and reverse transcribed as indicated above. The 2 × BlueStar™ PCR Master Mix kit was used for semi-quantitative RT-PCR analysis. One microliter of cDNA sample (about 400 ng/µL) was used for each independent PCR reaction using the following thermocycling conditions: 95 °C for 2 min, followed by 40 cycles of 98 °C for 10 s, 55 °C for 30 s, and a final extension at 72 °C for 60 s. The *DoMYB75* primer pair was the same as that used for vector construction. The primers UBQ10F/R for the *A*. *thaliana* ubiquitin gene (*AtUBQ10*) are listed in Supplementary Table 1.

### Analysis of water-soluble polysaccharide content

The WSPs in *D*. *officinale* stems were extracted and determined as previously described^34^. Whole mature and dry *A*. *thaliana* seeds were ground to a fine powder using a tissue lyser (TL2020, Beijing Haoyuan Technology Co. Ltd., Beijing, China). Twenty mg of powder was weighed precisely, pre-extracted twice with 1 mL 80% (v/v) hot ethanol for 20 min in each extraction step, and centrifuged by a Centrifuge 5424 R (Eppendorf, Hamburg, Germany) at 10,000 rpm for 10 min at 16 °C. The supernatant was discarded. The pellet was suspended with 2 mL of distilled water, then incubated in an ultrasonic bath (VCX600, Sonics and Materials Inc., Newtown, CT, USA) for 2 h at 60 °C to extract the WSPs. After centrifugation at 10,000 rpm for 10 min at 16 °C, the supernatant was collected and used to analyze WSPs by the phenol-sulfuric acid method^54^, as described in He *et al*.^34^.

### Statistical analyses

Data were analyzed using SigmaPlot12.3 software (Systat Software Inc., San Jose, CA, USA) by one-way analysis of variance (ANOVA) followed by Duncan’s multiple range test (DMRT) or Dunnett’s test. *P* < 0.05 was considered to be statistically significant.

## Results

### Identification of MYB superfamily genes in orchids

In this study, a total of 159 and 165 MYB TFs were identified in two orchids, *P*. *equestris* and *D*. *officinale*, respectively. In *P*. *equestris*, 40 MYBR genes, 115 R2R3-MYB genes, three 3R-MYB genes and one atypical MYB gene (CDC5-type) were found (Table 1). A similar number of MYB TF family members was found in *D*. *officinale*: 42 MYBR genes, 117 R2R3-MYB genes, four 3R-MYB genes and two atypical MYB genes (one 4RMYB and one CDC5-type) were identified in the *D*. *officinale* genome (Table 1). All the details of MYB genes in both orchids are listed in Supplementary Table 2.

**Table 1:** Number of members in the four groups of MYB transcription factors in *A*. *thaliana*, *P*. *equestris* and *D*. *officinale*.

Species	MYBR	2R-MYB	3R-MYB	Atypical MYB genes	Total
*A*. thaliana*	64	126	5	2	197
*P*. *equestris*	40	115	3	1	159
*D*. *officinale*	42	117	4	2	165

*The MYB genes from *A*. *thaliana* provided by Dubos *et al*.^10^ and Stracke *et al*.^74^.

### Classification of MYBR and R2R3-MYB proteins in two orchids

**Figure 1:**
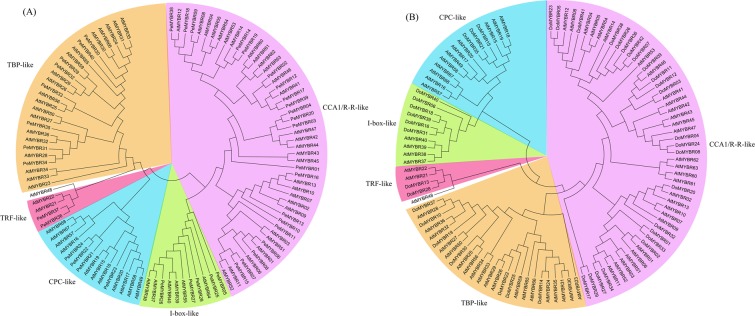
Phylogenetic trees of MYBR proteins. (**A**) Unrooted phylogenetic tree of *P. equestris* and *A. thaliana* MYBR proteins. (**B**) Unrooted phylogenetic tree of *D. officinale* and *A. thaliana* MYB proteins. The trees were generated by MEGA 7^44^ using the Neighbor-Joining method and aligned by MAFFT^42^.

In plants, MYBR proteins can be divided into five subgroups: CCA1/RR-like, CPC-like, I-box-like, TBP-like and TRF-like^2^. Genes in these five subgroups were also found in *P*. *equestris* and *D*. *officinale*, 21 and 24 CCA1/RR-like, 4 and 3 CPC-like, 5 and 6 I-box-like, 8 and 7 TBP-like, and 2 and 2 TRF-like, respectively. The CCA1/RR-like subfamily is the largest of the five subfamilies, while the TRF-like family is the smallest, with just two members in each orchid (Fig. 1A,B).

Compared with the MYB-related family, the R2R3-MYB family contained nearly three times as many genes as MYBR proteins in both orchids. The R2R3-MYB proteins are classified into 25 clades based on conservation of the MYB domain and C terminal amino acid motifs in *A*. *thaliana*^10^. To survey the classification within the R2R3-MYB gene family, we conducted a phylogenetic analysis of *A*. *thaliana* (126 members), *P. equestris* (115 members) and *D*. *officinale* (117 members) R2R3-MYB proteins. Based on a phylogenetic tree, all the MYB proteins could be grouped into 35 clades (C1-C35) (Table 2 and Supplementary Fig. 1). The C3 (S15), C4 (S5) and C13 (S23) clade genes were absent in both orchids, while C11 subfamily genes were only found in *P*. *equestris* and C17 clade genes were only present in the two orchids (Table 2 and Supplementary Fig. 1). This result suggests that the C3 (S15), C4 (S5) and C13 (S23) proteins might have been lost in orchids after divergence from the most recent common ancestor.

**Table 2:** Classification and putative functions of R2R3-MYB transcription factors.

Clade	*D*. *officinale*	*P*. *equestris*	*A*. *thaliana*	Functions in *A*. *thaliana*	References
C1 (*AtMYB5*)	*DoMYB01*	*PeMYB42*	*AtMYB5*	Testa mucilage synthesis	^25^
	*DoMYB06*	*PeMYB91*			
	*DoMYB42*	*PeMYB93*			
		*PeMYB95*			
	*DoMYB117*	*PeMYB104*	*AtMYB113*	Anthocyanin biosynthesis	^73^
C2 (S6)	*DoMYB74*	*PeMYB30*	*AtMYB114*	Anthocyanin biosynthesis	^73^
	*DoMYB75*	*PeMYB37*	*AtMYB75*	Cell wall thickening, testa, anthocyanin biosynthesis	^70– 72^
	*DoMYB86*	*PeMYB54*	*AtMYB90*		
			*AtMYB0*		
C3 (S15)			*AtMYB23*	Trichome morphogenesis	^75^
			*AtMYB66*		
C4 (S5)			*AtMYB123*		
	*DoMYB22*	*PeMYB40*	*AtMYB108*	Stamen maturation	^76^
	*DoMYB96*	*PeMYB75*	*AtMYB112*	Flavonoid biosynthesis	^77^
C5 (S20)		*PeMYB86*	*AtMYB116*		
		*PeMYB87*	*AtMYB2*	Phosphate-starvation responses	^78^
			*AtMYB62*	Phosphate-starvation responses	^79^
			*AtMYB78*		
C6 (S19)	*DoMYB44*	*PeMYB18*	*AtMYB21*		
	*DoMYB62*	*PeMYB70*	*AtMYB24*	Stamen maturation	^76^
	*DoMYB108*	*PeMYB02*	*AtMYB121*		
	*DoMYB34*	*PeMYB27*	*AtMYB27*		
	*DoMYB53*	*PeMYB34*	*AtMYB48*		
C7 (S17)	*DoMYB66*	*PeMYB41*	*AtMYB59*	Root growth; cell cycle progression	^80^
	*DoMYB77*	*PeMYB44*	*AtMYB71*		
	*DoMYB91*	*PeMYB49*			
		*PeMYB94*			
	*DoMYB02*	*PeMYB01*	*AtMYB105*		
	*DoMYB111*	*PeMYB04*	*AtMYB110*		
	*DoMYB16*	*PeMYB26*	*AtMYB117*		
	*DoMYB26*	*PeMYB32*	*AtMYB52*	Secondary cell wall biosynthesis	^81^
	*DoMYB27*	*PeMYB36*	*AtMYB54*	Secondary cell wall biosynthesis	^81^
C8 (S21)	*DoMYB31*	*PeMYB45*	*AtMYB56*		
	*DoMYB36*	*PeMYB48*	*AtMYB69*		
	*DoMYB45*	*PeMYB64*			
	*DoMYB52*	*PeMYB65*			
	*DoMYB64*	*PeMYB67*			
	*DoMYB88*	*PeMYB80*			
	*DoMYB97*	*PeMYB82*			
	*DoMYB98*	*PeMYB84*			
C9 (*AtMYB124*/*AtMYB88*)	*DoMYB106*	*PeMYB29*	*AtMYB124*		
			*AtMYB88*		
C10 (*AtMYB91*)	*DoMYB18*	*PeMYB57*	*AtMYB91*		
		*PeMYB105*			
		*PeMYB106*			
C11		*PeMYB112*			
		*PeMYB115*			
		*PeMYB89*			
	*DoMYB03*	*PeMYB31*	*AtMYB44*	Root development	^82^
	*DoMYB24*	*PeMYB38*	*AtMYB77*		
C12 (S22)	*DoMYB30*	*PeMYB73*	*AtMYB70*		
	*DoMYB84*		*AtMYB73*		
	*DoMYB94*				
			*AtMYB1*	Salt stress response	^83^
C13 (S23)			*AtMYB109*		
			*AtMYB35*		
C14 (*AtMYB125*)	*DoMYB114*	*PeMYB09*	*AtMYB125*		
	*DoMYB70*	*PeMYB88*	*AtMYB36*	Root development	^84^
	*DoMYB109*	*PeMYB110*	*AtMYB100*		
	*DoMYB13*	*PeMYB28*	*AtMYB115*	Benzoyloxy glucosinolate pathway	^85^
C15 (S25)	*DoMYB15*	*PeMYB90*	*AtMYB118*	Benzoyloxy glucosinolate pathway	^86^
	*DoMYB83*		*AtMYB119*	Gametogenesis	^86^
	*DoMYB87*		*AtMYB22*		
			*AtMYB64*	Gametogenesis	^86^
	*DoMYB107*	*PeMYB107*	*AtMYB101*	Fertilization	^87^
	*DoMYB12*	*PeMYB114*	*AtMYB104*		
	*DoMYB25*	*PeMYB23*	*AtMYB120*	Fertilization	^87^
C16 (S18)	*DoMYB50*	*PeMYB59*	*AtMYB33*		
	*DoMYB55*		*AtMYB65*		
	*DoMYB72*		*AtMYB81*		
			*AtMYB97*	Fertilization	^87^
C17	*DoMYB73*	*PeMYB50*			
		*PeMYB83*			
	*DoMYB04*	*PeMYB05*	*AtMYB36*		
	*DoMYB11*	*PeMYB08*	*AtMYB37*		
	*DoMYB35*	*PeMYB102*	*AtMYB38*		
	*DoMYB38*	*PeMYB103*	*AtMYB68*		
	*DoMYB49*	*PeMYB12*	*AtMYB84*		
C18 (S14)	*DoMYB56*	*PeMYB13*	*AtMYB87*	Cell wall organization	^88^
	*DoMYB68*	*PeMYB46*			
	*DoMYB79*	*PeMYB51*			
	*DoMYB89*	*PeMYB53*			
	*DoMYB95*	*PeMYB66*			
		*PeMYB74*			
		*PeMYB76*			
C19 (*AtMYB80*/*AtMYB35*)	*DoMYB102*	*PeMYB24*	*AtMYB80*		
	*DoMYB113*	*PeMYB69*	*AtMYB35*		
	*DoMYB19*				
	*DoMYB05*	*PeMYB33*	*AtMYB30*		
	*DoMYB37*	*PeMYB78*	*AtMYB31*		
C20 (S1)	*DoMYB57*	*PeMYB97*	*AtMYB60*		
		*PeMYB98*	*AtMYB94*	Cuticular wax biosynthesis	^89^
			*AtMYB96*	Cuticular wax biosynthesis	^89^
	*DoMYB23*	*PeMYB15*	*AtMYB107*	Suberin deposition	^90^
C21 (S10)	*DoMYB47*	*PeMYB22*	*AtMYB39*		
	*DoMYB82*		*AtMYB9*	Suberin deposition	^90^
	*DoMYB104*	*PeMYB79*	*AtMYB53*		
C22 (S24)	*DoMYB61*		*AtMYB92*		
	*DoMYB76*		*AtMYB93*	Root development	^91^
	*DoMYB39*	*PeMYB21*	*AtMYB102*		
	*DoMYB40*	*PeMYB99*	*AtMYB41*	Osmotic stress responses	^92^
C23 (S11)	*DoMYB41*		*AtMYB74*	Salt stress responses	^93^
	*DoMYB51*				
	*DoMYB58*				
	*DoMYB32*	*PeMYB25*	*AtMYB106*		
C24 (S9)	*DoMYB60*	*PeMYB52*	*AtMYB16*	Cuticle formation	^94^
	*DoMYB69*	*PeMYB56*	*AtMYB17*		
	*DoMYB100*	*PeMYB06*	*AtMYB122*		
C25 (S12)	*DoMYB43*		*AtMYB51*		
			*AtMYB34*		
			*AtMYB29*		
			*AtMYB76*		
	*DoMYB10*	*PeMYB03*	*AtMYB13*		
	*DoMYB28*	*PeMYB109*	*AtMYB14*	Testa polymer biosynthesis	^25^
C26 (S2)	*DoMYB54*	*PeMYB47*	*AtMYB15*		
	*DoMYB71*				
	*DoMYB78*				
	*DoMYB99*				
	*DoMYB46*	*PeMYB14*	*AtMYB58*	Secondary cell wall biosynthesis	^95^
C27 (S3)	*DoMYB92*	*PeMYB19*	*AtMYB63*	Secondary cell wall biosynthesis	^95^
		*PeMYB85*			
	*DoMYB116*	*PeMYB113*	*AtMYB11*	Flavonoid biosynthesis	^96^
G28 (S7)	*DoMYB59*	*PeMYB43*	*AtMYB111*	Flavonoid biosynthesis	^96^
		*PeMYB68*	*AtMYB12*	Flavonoid biosynthesis	^96^
C29 (*AtMYB47*/*AtMYB95*)	*DoMYB20*	*PeMYB63*	*AtMYB47*		
			*AtMYB95*		
	*DoMYB07*	*PeMYB17*	*AtMYB20*		
	*DoMYB103*	*PeMYB20*	*AtMYB40*	Secondary cell wall biosynthesis	^81^
C30 (S8)	*DoMYB67*	*PeMYB62*	*AtMYB42*	Secondary cell wall biosynthesis	^81^
		*PeMYB96*	*AtMYB43*	Secondary cell wall biosynthesis	^81^
			*AtMYB85*	Secondary cell wall biosynthesis	^81^
			*AtMYB99*		
	*DoMYB21*	*PeMYB07*	*AtMYB3*	Phenylpropanoid biosynthesis	^97^
	*DoMYB33*	*PeMYB35*	*AtMYB32*		
C31 (S4)	*DoMYB48*	*PeMYB39*	*AtMYB4*		
	*DoMYB80*	*PeMYB58*	*AtMYB7*		
	*DoMYB85*	*PeMYB60*			
	*DoMYB93*				
	*DoMYB08*		*AtMYB18*		
C32 (S16)			*AtMYB19*		
			*AtMYB45*		
	*DoMYB09*	*PeMYB100*	*AtMYB50*		
	*DoMYB110*	*PeMYB101*	*AtMYB55*		
	*DoMYB112*	*PeMYB108*	*AtMYB61*	Testa mucilage synthesis	^26, 98^
	*DoMYB115*	*PeMYB11*	*AtMYB86*		
C33 (S13)	*DoMYB14*	*PeMYB111*			
	*DoMYB29*	*PeMYB55*			
	*DoMYB65*	*PeMYB71*			
	*DoMYB81*	*PeMYB77*			
		*PeMYB81*			
C34 (*AtMYB26*/*AtMYB67*/*AtMYB103*)	*DoMYB105*	*PeMYB16*	*AtMYB26*	Secondary cell wall thickening	^99^
	*DoMYB63*	*PeMYB61*	*AtMYB67*		
	*DoMYB90*	*PeMYB72*	*AtMYB103*	Lignin biosynthesis; secondary cell wall thickening	^100^
		*PeMYB92*			
C35 (AtMYB46/AtMYB83)	*DoMYB17*	*PeMYB10*	*AtMYB46*	Secondary wall biosynthesis	^69, 101^
			*AtMYB83*	Secondary wall biosynthesis	^69^

### Non-synonymous (Ka) and synonymous (Ks) substitutions in orthologous gene pairs between *P*. *equestris* and *D*. *officinale*

The Ka/Ks value is regarded as a pointer to assess selective pressure on a protein-coding gene. A Ka/Ks ratio less than 1 indicates a negative or purifying selection, a Ka/Ks ratio equal to 1 indicates neutral evolution, while a Ka/Ks ratio greater than 1 indicates positive or adaptive evolution. In total, 84 orthologous gene pairs between *P*. *equestris* and *D*. *officinale* were found (Table 3). In other words, about 50% of orchid MYB genes appeared to be duplicated. This suggests that most orchid MYB genes underwent functional diversity and expansion during evolution. In our research, most of these orthologous gene pairs were deduced to be under negative selection with a Ka/Ks ratio less than 1, except for *DoMYBR22* and *PeMYBR29*, which had a Ka/Ks ratio greater than 1 (Table 3).

**Table 3:** Ka/Ks analysis and estimated selective pressure for orthologous gene pairs between *P*. *equestris* and *D*. *officinale*.

Paralogous gene pairs		Ka	Ks	Ka/Ks	*P*-Value (Fisher)
*D*. *officinale*	*P*. *equestris*				
DoMYBR22	PeMYBR29	0.252639	0.226031	1.11772	0.359576
DoMYBR01	PeMYBR11	0.229767	0.264664	0.868147	4.37E-01
DoMYB30	PeMYBCDC	0.540245	0.678633	0.796079	3.34E-01
DoMYB113	PeMYB24	0.281005	0.395265	0.710927	9.42E-02
DoMYBR35	PeMYBR22	0.174475	0.302301	0.577156	1.47E-01
DoMYBR02	PeMYBR06	0.187853	0.333361	0.563513	1.95E-05
DoMYBR28	PeMYBR37	0.288114	0.517813	0.556406	0.002783
DoMYBR30	PeMYBR33	0.587592	1.18628	0.495324	2.48E-05
DoMYBR33	PeMYBR08	0.14577	0.324747	0.448873	4.66E-08
DoMYB50	PeMYB108	0.1481	0.330785	0.447722	8.97E-07
DoMYB94	PeMYB73	0.181577	0.411152	0.441631	0.001206
DoMYB35	PeMYB102	0.129134	0.294916	0.437867	0.000129
DoMYB27	PeMYB80	0.135674	0.311095	0.436116	3.87E-05
DoMYB727	PeMYB23	0.268111	0.652068	0.41117	5.06E-09
DoMYB22	PeMYB40	0.195574	0.487158	0.401458	1.66E-05
DoMYBR25	PeMYBR32	0.174571	0.461248	0.378476	4.33E-08
DoMYB20	PeMYB63	0.216538	0.5768	0.375412	9.05E-06
DoMYB106	PeMYB29	0.124663	0.350774	0.355394	3.95E-09
DoMYBR13	PeMYBR36	0.160839	0.461041	0.34886	8.74E-08
DoMYB65	PeMYB11	0.127844	0.376824	0.339266	1.8E-10
DoMYBR14	PeMYBR30	0.148252	0.460324	0.322059	1.47E-18
DoMYB02	PeMYB26	0.139964	0.434743	0.321947	2.6E-07
DoMYB46	PeMYB14	0.143144	0.445163	0.321555	3.64E-09
DoMYB87	PeMYB28	0.172107	0.540112	0.31865	6.7E-10
DoMYB3R1	PeMYB3R1	0.115854	0.367231	0.31548	4.86E-14
DoMYB63	PeMYB61	0.1622	0.485588	0.30112	7.53E-11
DoMYB97	PeMYB67	0.122021	0.419452	0.290905	4.14E-09
DoMYBR32	PeMYBR10	0.07528	0.263304	0.285905	3.16E-08
DoMYBR20	PeMYBR16	0.078868	0.286058	0.275706	6.34E-11
DoMYB95	PeMYB66	0.099102	0.362533	0.273361	2.15E-08
DoMYB52	PeMYB32	0.156645	0.578849	0.270614	4.84E-12
DoMYB36	PeMYB48	0.123647	0.465682	0.265519	1.93E-07
DoMYBR15	PeMYBR21	0.237202	0.901119	0.263231	0.003537
DoMYB06	PeMYB91	0.131802	0.517651	0.254616	1.24E-10
DoMYBR19	PeMYBR35	0.077925	0.310476	0.250985	2.15E-10
DoMYB09	PeMYB81	0.100041	0.398857	0.250819	8.1E-10
DoMYBR09	PeMYBR12	0.098051	0.392184	0.250012	1.16E-12
DoMYB77	PeMYB34	0.099167	0.399335	0.248331	4.09E-09
DoMYBR40	PeMYBR01	0.125086	0.51066	0.24495	4.89E-06
DoMYB91	PeMYB41	0.079636	0.333581	0.238729	2.76E-08
DoMYB38	PeMYB53	0.095368	0.4069	0.234376	7.07E-14
DoMYB04	PeMYB74	0.083609	0.36295	0.230358	4.17E-10
DoMYB43	PeMYB06	0.109957	0.48052	0.228829	3.95E-13
DoMYB68	PeMYB51	0.072732	0.325264	0.223608	3.18E-10
DoMYBR10	PeMYBR31	0.07664	0.349733	0.219138	4.03E-08
DoMYB111	PeMYB45	0.093899	0.434686	0.216015	2.4E-08
DoMYB19	PeMYB69	0.092868	0.459706	0.202017	2.53E-16
DoMYB92	PeMYB85	0.105135	0.526037	0.199862	1.51E-13
DoMYB108	PeMYB44	0.092366	0.469872	0.196577	1.14E-12
DoMYBR17	PeMYBR05	0.060215	0.308167	0.195397	1.47E-21
DoMYBR11	PeMYBR02	0.093764	0.48181	0.194608	1.11E-15
DoMYB26	PeMYB04	0.132161	0.687219	0.192312	6.56E-12
DoMYBR24	PeMYBR20	0.061053	0.343438	0.177769	5.88E-16
DoMYB23	PeMYB22	0.078265	0.460628	0.16991	3.87E-21
DoMYB07	PeMYB20	0.060264	0.356788	0.168906	1.25E-13
DoMYB84	PeMYB31	0.199724	1.27411	0.156756	5.73E-22
DoMYB59	PeMYB114	0.125627	0.887715	0.141517	1.47E-17
DoMYB13	PeMYB90	0.123877	0.879398	0.140866	8.44E-18
DoMYB17	PeMYB10	0.103123	0.733765	0.14054	1.39E-24
DoMYB01	PeMYB42	0.09443	0.685337	0.137786	5.07E-19
DoMYB96	PeMYB86	0.062697	0.46096	0.136014	2.21E-19
DoMYBR37	PeMYBR34	0.05965	0.454563	0.131225	6.45E-23
DoMYB57	PeMYB33	0.044033	0.349604	0.125952	1.68E-16
DoMYB64	PeMYB36	0.049998	0.39719	0.12588	1.82E-17
DoMYB66	PeMYB02	0.093345	0.755975	0.123477	3.24E-21
DoMYB24	PeMYB38	0.109397	0.886578	0.123393	3.53E-32
DoMYBR03	PeMYBR17	0.067015	0.544493	0.123078	2.42E-25
DoMYBR31	PeMYBR26	0.098098	0.816452	0.120151	1.76E-10
DoMYB61	PeMYB79	0.058864	0.495516	0.118792	1.14E-22
DoMYB103	PeMYB62	0.064227	0.566367	0.113402	4.68E-22
DoMYBR39	PeMYBR28	0.060879	0.555284	0.109635	1.13E-08
DoMYB51	PeMYB21	0.042387	0.400007	0.105965	2.99E-26
DoMYB18	PeMYB57	0.064086	0.618519	0.103613	5.75E-32
DoMYB47	PeMYB15	0.061099	0.602174	0.101465	7.01E-31
DoMYB34	PeMYB49	0.072019	0.786213	0.091603	5.96E-25
DoMYB69	PeMYB56	0.071401	0.785038	0.090952	1.5E-34
DoMYB44	PeMYB18	0.036016	0.429379	0.083879	1.45E-18
DoMYB112	PeMYB99	0.218163	2.74748	0.079405	2.25E-12
DoMYB12	PeMYB107	0.231365	3.25787	0.071017	5.2E-11
DoMYBR06	PeMYBR25	0.037052	0.562053	0.065922	1.61E-12
DoMYB09	PeMYB16	0.039861	0.637587	0.062519	5.85E-34
DoMYB80	PeMYB35	0.045443	0.806638	0.056336	7.78E-28
DoMYB116	PeMYB113	0.136094	2.97652	0.045722	4.68E-23

Ka non-synonymous substitutions per non-synonymous site, Ks synonymous substitutions per synonymous site); Ka/Ks the ratio.

### *Cis*-responsive element analysis of MYB genes from *P*. *equestris* and *D*. *officinale*

**Figure 2:**
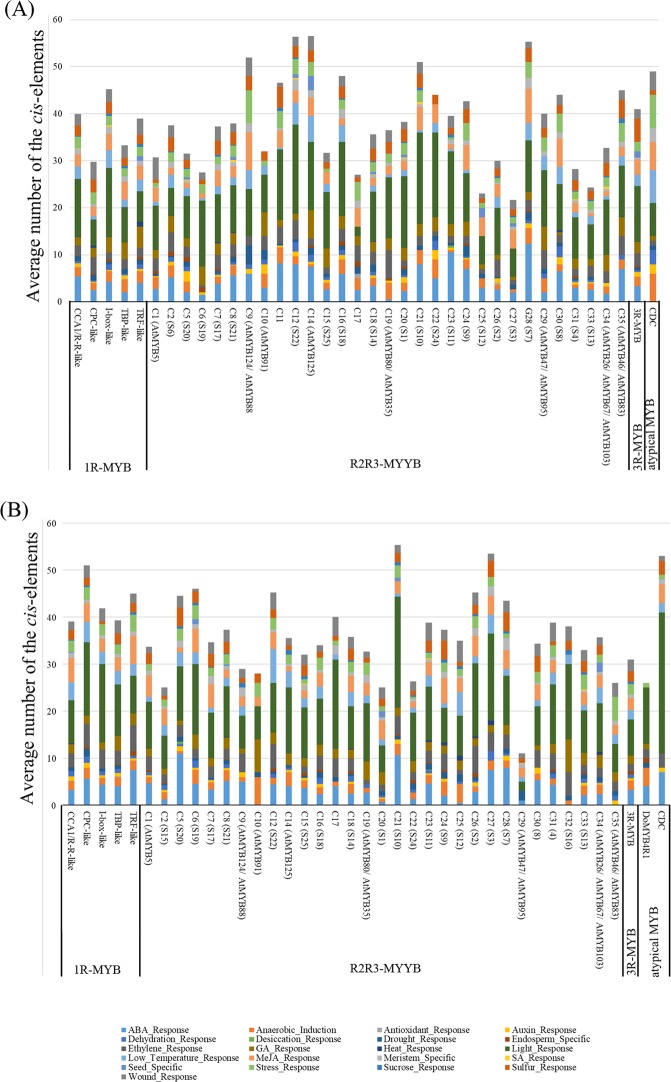
Average number of *cis*-responsive elements of orchid MYB genes from each group. The *cis*-responsive elements were analyzed in the 2 kb upstream promoter region of the initiation codon using the PlantCARE database and the PLACE database.

All the 2000 bp upstream regions of the initiation codon of MYB genes from *P*. *equestris* and *D*. *officinale* were obtained from their respective genomes. The stress response elements, tissue-specific activation, hormone responsive elements, and other responsive elements were identified and analyzed (Fig. 2). Various stress responsive elements, including anaerobic induction, and response to antioxidant, dehydration, desiccation, drought, heat, low temperature, stress, and wound elements, were analyzed. Only anaerobic induction, low temperature response and wound response elements were widely present in the MYB gene promoters of both orchids (Fig. 2 and Supplementary Table 3). Hormone responsive elements such as ABA, ethylene, GA and MeJA response were abundant in the putative promoters of MYB genes, especially the ABA response element (Fig. 2). ABA, ethylene and MeJA are related to stress response in plants^55^. These results suggested that the MYB genes may play an important role in stress response in orchids.

### Expression analyses of MYB genes under cold stress in *D*. *officinale*

**Figure 3:**
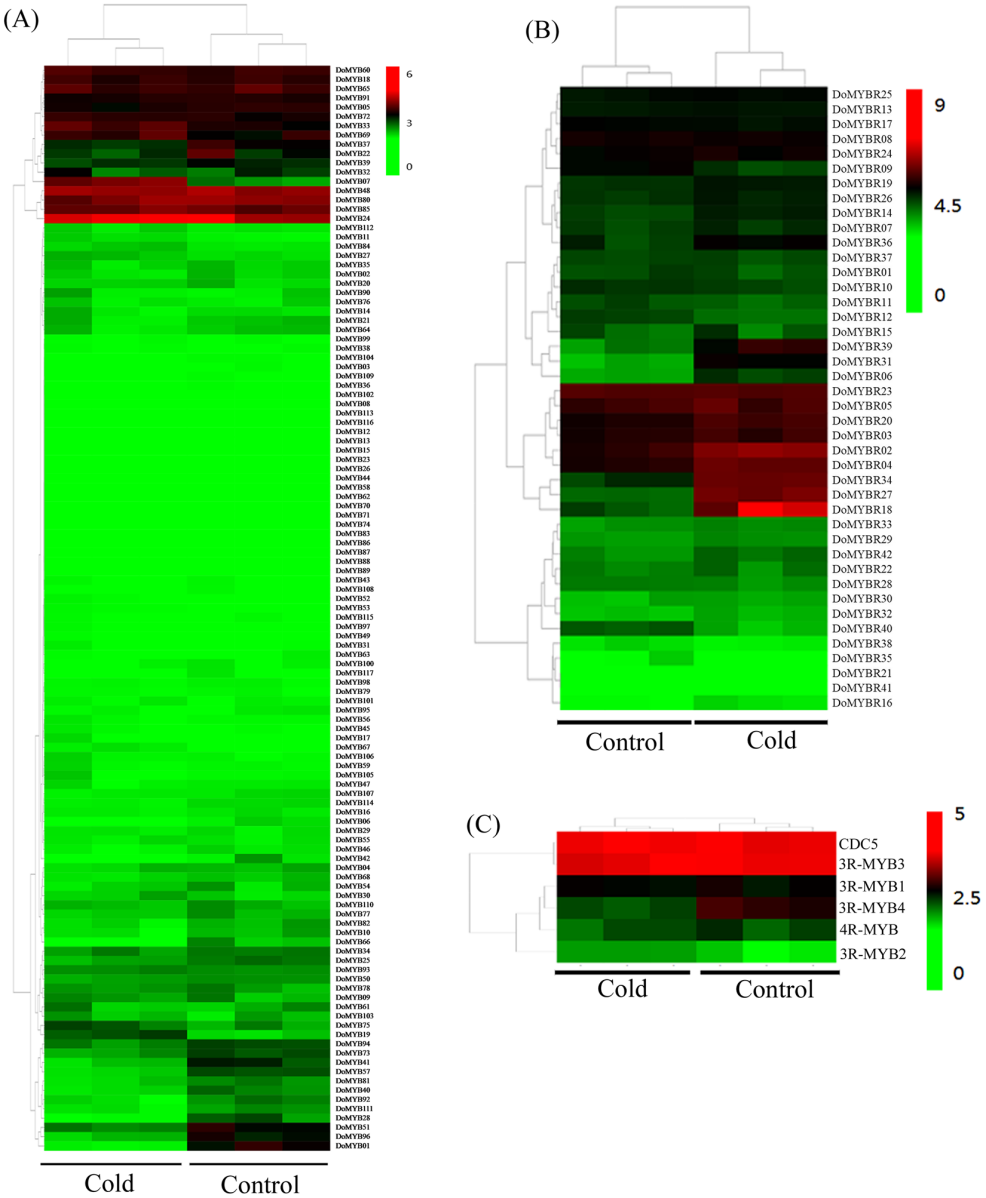
Expression patterns of the four groups of MYB genes from *D. officinale* under cold stress (4 °C). Expression profiles of R2R3-MYB genes (**A**), MYBR genes (**B**), and 3R-MYB and atypical MYB genes (**C**). The heatmap was generated using R version 3.4.1 with a color scale according to the gene expression level [log_2_ (FPKM + 1)]. Red indicates high gene expression level while green indicates a low level of expression. Each column indicates a discrete biological sample. All treatments consisted of three biological replicates.

MYB genes are involved in a plant’s response to stress, such as drought and cold stress^56–58^. To gain insight into *D*. *officinale* MYB proteins in stress responses, the expression of MYB genes were evaluated under the control condition (20 °C) and cold stress (4 °C) by comparing the FPKM values for each gene at 20 °C and 4 °C. Only nine of 117 R2R3-MYB genes were modulated by cold stress, consisting of three up-regulated genes (*DoMYB07*, −*33*, and −*69*) and six down-regulated genes (*DoMYB01*, −*22*, −*37*, −*41*, −*51*, and −*96*) (Fig. 3A and Supplementary Table 4). A total of 15 out of the 42 MYBR genes (*DoMYBR02*, −*04*, −*06*, −*09*, −*12*, −*14*, −*18*, −*20*, −*27*, −*31*, −*34*, −*36*, −*39*, −*40*, and −*42*) were modulated by cold stress, nine of which were up-regulated by at least two-fold while three genes were down-regulated (less than 0.5-fold, Fig. 3B). Four up-regulated genes (*DoMYBR06*, −*18*, −*31*, and −*39*) were from the I-box-like subfamily, seven up-regulated genes (*DoMYBR02*, −*04*, −*20*, −*27*, −*34*, −*36* and −*42*) were from the CCA1/R-R-like subfamily, and one gene (*DoMYBR14*) was from the TBP-like clade (Fig. 3 and Supplementary Table 4). Three 3R-MYBs, all 4R-MYBs and only one CDC5-type genes of *D*. *officinale* showed no differences between the control and cold stress (Fig. 3 and Supplementary Table 4). One 3R-MYB (*DoMYB3R4*) gene was down-regulated (Fig. 3 and Supplementary Table 4).

### Identification and expression analysis of R2R3-MYB genes related to polysaccharide biosynthesis

Polysaccharides are abundant in the secondary cell wall and testa mucilage in *A*. *thaliana*^24,59^. Members of nine clades, namely C1 (AtMYB5), C2 (S6), C8 (S21), C26 (S2), C27 (S3), C30 (S8), C33 (S13), C34 (AtMYB26/AtMYB67/AtMYB103) and C35 (AtMYB46/AtMYB83), are involved in secondary cell wall or testa mucilage biosynthesis in *A*. *thaliana* (Table 2). The genes from these clades are probably involved in polysaccharide biosynthesis and are regarded as putative MYB genes of polysaccharide biosynthesis. A total of 32, 45 and 43 putative MYB polysaccharide biosynthesis genes were identified from *A*. *thaliana*, *P*. *equestris* and *D*. *officinale*, respectively.

**Figure 4:**
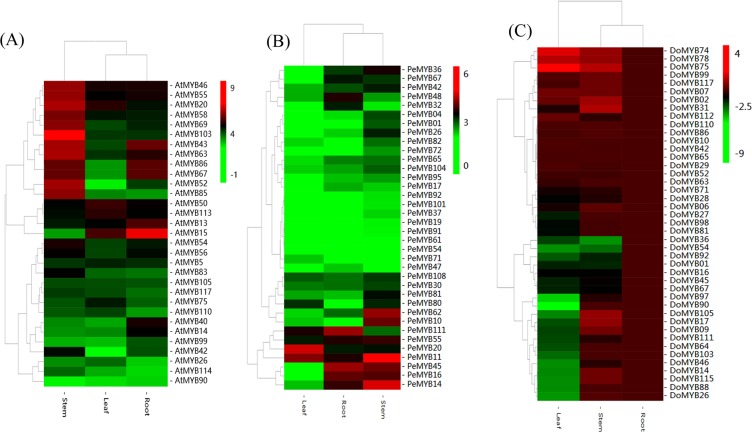
Heat map displaying the expression pattern of nine clades of R2R3-MYB genes in roots, stems and leaves. (**A**) Expression profile of 32 R2R3-MYB genes in different tissues or organs of *A. thaliana* from microarray data sets available at Gene Expression Omnibus (GEO, https://www.ncbi.nlm.nih.gov/geo/). GEO accessions included: GSM131558, GSM131559 and GSM131560 (roots); GSM131655, GSM131656 and GSM131657 (stems); GSM131528, GSM131529 and GSM131530 (leaves). The color scale represents log_2_ of the mean of gene expression. (**B**) Expression profile of 36 R2R3-MYB genes from *P. equestris*. Expression data were downloaded from Orchidbase (http://orchidbase.itps.ncku.edu.tw). The color scale was based on the gene expression level, which was measured as log_2_ (gene expression valuen + 1). (**C**) Analysis of the 43 R2R3-MYB genes from *D. officinale* based on an analysis of qRT-PCR results, and visualized as a heatmap. The color scale represents log2 of the mean of gene expression. At least two biological replicates were performed.

To comparatively analyze their expression patterns, the mRNA steady state levels of the putative MYB genes related to polysaccharide biosynthesis were monitored in the roots, stems and leaves of *A*. *thaliana*, *P*. *equestris* and *D*. *officinale*. Interestingly, 13 of the putative MYB genes from *A*. *thaliana* (*AtMYB20*, −*42*, −*43*, −*46*, −*52*, −*54*, −*55*, −*58*, −*63*, −*69*, −*83*, −*85*, and −*103*) were highly expressed in stems, and most of these genes were also involved in secondary cell wall biosynthesis (Fig. 4A, Supplementary Table 5). Eight (*PeMYB10*, −*11*, −*14*, −*26*, −*36*, −*42*, −*62*, and −*81*) and nine (*DoMYB02*, −*09*, −*14*, −*17*, −*31*, −*99*, −*105*, −*115*, and −*117*) MYB genes were highly expressed in the stems of *P*. *equestris* and *D. officinale*, respectively (Fig. 4B,C, Supplementary Tables 5 and 6). All C35 (AtMYB46/AtMYB83) genes were abundantly expressed in stems (Fig. 4).

### Screening R2R3-MYB genes involved in biosynthesis of water-soluble polysaccharides in *D*. *officinale* and their expression under water deficit stress

**Figure 5:**
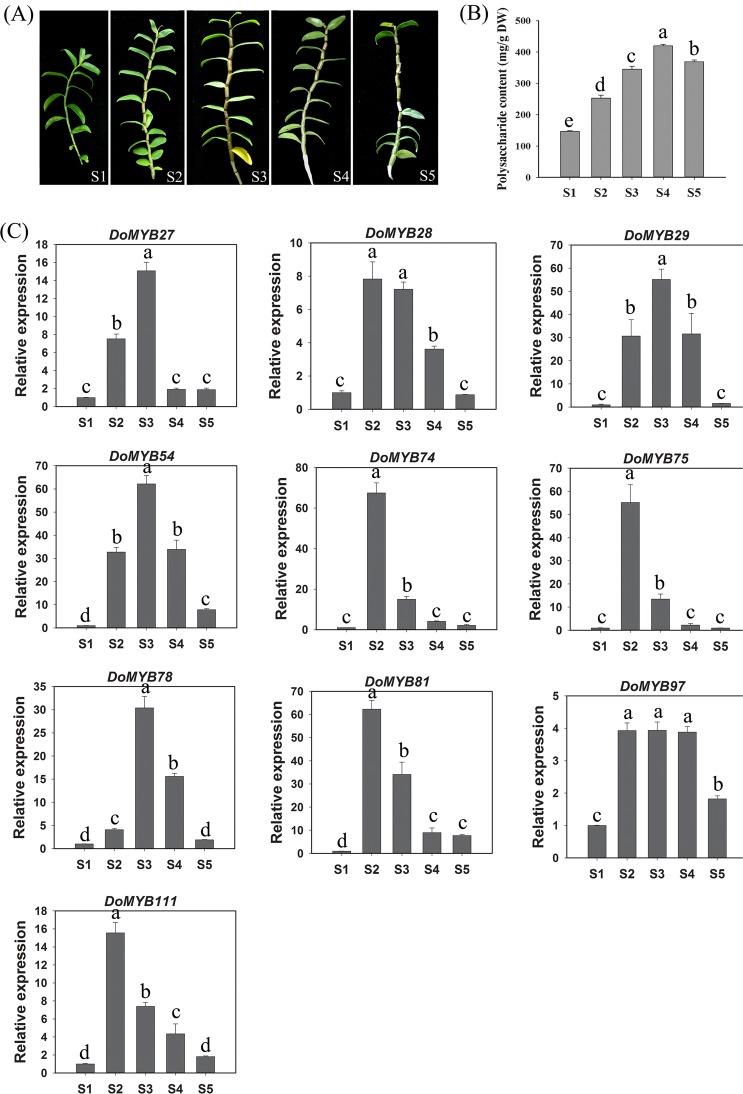
Screening candidate R2R3-MYB genes related to the biosynthesis of WSPs in *D. officinale* by qRT-PCR. (**A**) Five stages of *D. officinale* stems were used to analyze gene expression. (**B**) Polysaccharide content in the stems of five stages. DW, dry weight. (**C**) Candidate genes identified using qRT-PCR. Details pertaining to S1-S5 can be found in the materials and methods and results sections. Bars represent mean ± SD (n = 3). Three biological replicates were performed. Different letters in each bar are significantly different at *P* < 0.05 (Duncan’s multiple range test).

*D*. *officinale* is one of the most precious Chinese herbs with abundant WSPs in its stems^33^. The accumulation of WSPs in stems changes with plant growth^34^. To further screen the genes involved in the biosynthesis of WSPs, the expression of the 43 *D*. *officinale* genes belonging to the clusters C1 (AtMYB5), C2 (S6), C8 (S21), C26 (S2), C27 (S3), C30 (S8), C33 (S13), C34 (AtMYB26/AtMYB67/AtMYB103) and C35 (AtMYB46/AtMYB83) was analyzed across five plant developmental stages. In the S1 stage, plants were in the vegetative stage and had lowest WSP content (Fig. 5A,B). In stages S2-4, plants stopped growing and accumulated WSPs rapidly (Fig. 5A,B). In the S5 stage, plants started to undergo senescence and degradation of WSPs (Fig. 5A,B).

Ten genes, out of the 43 tested, showed an expression pattern that mirrored the accumulation pattern of WPS. Among these genes, six peaked at S2 (*DoMYB28*, −*74*, −*75*, −*81*, −*97* and −*111*) and four at S3 (*DoMYB27*, −*29*, −*54* and −*78*). All these genes were poorly expressed at S1, and most of them at S5 returned to the levels of S1 (Fig. 5C). The 10 MYB genes included two (*DoMYB74* and −75) from C2 (S6), three (*DoMYB27*, −97, and −*111*) from C8 (S21), three (*DoMYB28*, −*54*, and −*78*) from C26 (S2) and two (*DoMYB29* and −*81*) from C33 (S13). The remaining genes were either not detected or were inconsistent with changes in polysaccharide content (Supplementary Fig. 2).

**Figure 6:**
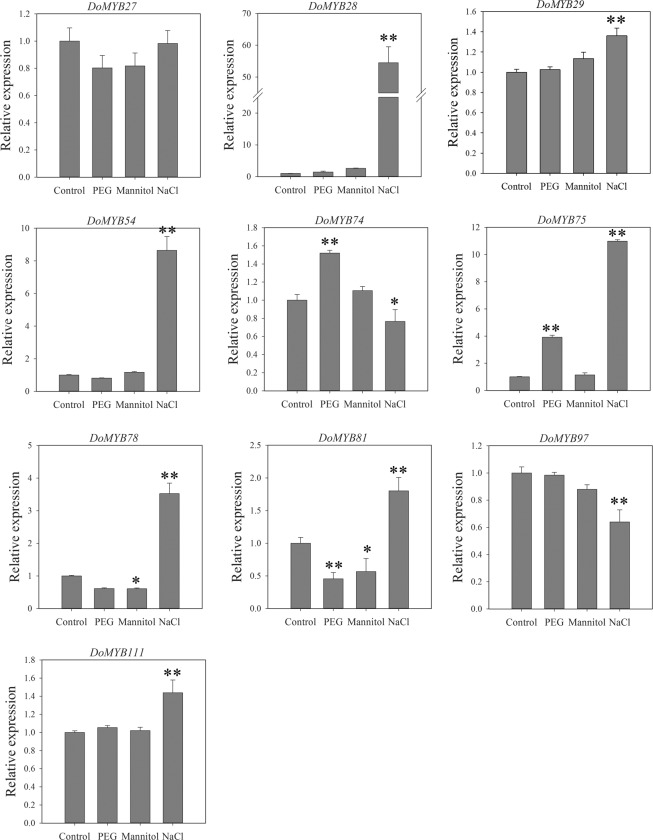
Analysis of the expression level of ten R2R3-MYB genes by qRT-PCR after *D*. *officinale* seedlings were subjected to PEG (150 g/L), mannitol (300 mM) and salt (NaCl, 250 mM) treatments. Control are seedlings treated with ½MS medium supplemented with 20 g/L sucrose (pH 5.4). Bars represent mean ± SD of three technical replicates. Three biological replicates of each treatment were performed. *indicates *P* < 0.05; **indicates *P* < 0.001 between control and stress treatments following Dunnett’s test.

In our previous studies in *D*. *officinale*, the genes of the WSP biosynthetic pathway were shown to be involved in abiotic stresses influenced by PEG and NaCl treatment^34,36^. Thus, the expression of candidate genes under 150 g/L PEG, 300 mM mannitol and 250 mM NaCl treatments was analyzed. Our results indicate that most of the candidate genes were up-regulated under salinity stress, i.e. *DoMYB28*, −*29*, −*54*, −*75*, −*78*, −*81* and −*111*. Only two genes (*DoMYB74* and −*75*) were up-regulated after exposure to PEG while only one gene (*DoMYB81*) was down-regulated after exposure to both PEG and mannitol (Fig. 6). *DoMYB27* expression showed no significant difference between stress treatments and the control (Fig. 6). *DoMYB75* was up-regulated in response to PEG and NaCl, similar with other WSP biosynthetic pathway genes such as *DoPMM*^36^ and *DoCSLAs*^34^.

### Overexpression of *DoMYB75* in *A*. *thaliana* confirms its role in the biosynthesis of water-soluble polysaccharides

**Figure 7:**
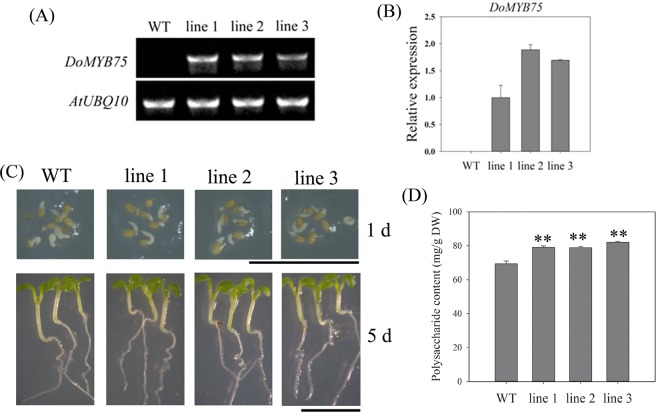
Characterization of the *DoMYB75* gene in the biosynthesis of WSPs. (**A**) Analysis of the *DoMYB75* gene in wild type (WT) and transgenic lines by semi-quantitative PCR. (**B**) Analysis of the *DoMYB75* gene in WT and transgenic lines by qRT-PCR. Expression levels were calculated relative to transgenic line 1. (**C**) Germinating seeds (one day after stratification) and seedlings (5 days after stratification) of WT and transgenic lines showed no obvious phenotypic changes. (**D**) Content of WSPs in mature dry seeds of *A. thaliana*. **Indicates *P* < 0.01 between WT and transgenic lines following Dunnett’s test.

*DoMYB75* may be involved in WSP biosynthesis, so it was used in further analyses. In a phylogenetic tree, the C2 (S6) clade was divided into two branches: cluster I included proteins of monocots (*P*. *equestris* and *D*. *officinale*) while cluster II only contained the proteins of *A*. *thaliana*, a dicot (Supplementary Fig. 3A). The complete CDS of *DoMYB75* without a termination codon was cloned into an over-expression vector (pCABIA 1302 vector) driven by the CaMV-35S promoter (Supplementary Fig. 3B). *DoMYB75* was detected and expressed in *DoMYB75* transgenic Arabidopsis lines, but not in WT plants (Fig. 7A,B). Overexpression of *DoMYB75* showed no difference in germinating seeds or seedlings (Fig. 7C). The average WSP content of seeds of all transgenic plants (around 79 mg/g DW) was significantly higher than WT plants (~69 mg/g DW, Fig. 7D). This indicates that *DoMYB75* plays a role in WSP biosynthesis in *A*. *thaliana* seeds.

## Discussion

### Identification and classification of MYB proteins

In this study, we identified 159 and 165 MYB genes from *P*. *equestris* and *D*. *officinale* genomes, respectively. Although these orchid species belong to different genera, the number of MYB genes in their genomes is similar. Four groups of MYB proteins were found in *D*. *officinale*, similar to previous studies in other plant species such as *A*. *thaliana*^6^, *G*. *max*^2,13^ and *P*. *bretschneideri*^8^. However, no 4R-MYB gene was found in the genome of *P*. *equestris*, which had only one atypical MYB gene, namely the CDC5-type gene. Another monocot, rice, contained one CDC5-type gene but not a 4R-MYB gene in its genome^11^, similar to *P*. *equestris* in our study. This suggests that 4R-MYB genes are not found in all higher plants and might not play essential roles in all plants.

The MYBR proteins in both orchids could be divided into five subfamilies, with CCA1/RR-like as the largest subfamily, and containing two TRB-like genes in their genomes. Five MYBR subfamilies were also found in other higher plants such as *A*. *thaliana*, *S*. *lycopersicum*, *V*. *vinifera*, and *B*. *distachyon*, with the number of CCA1/RR-like genes ranging from 21 to 42, and with all plants just containing only one or two TRF-like genes in their genomes^2^. The R2R3-MYB group is largest group of the MYB family, with more than 100 members having been found in the genomes of both monocots and dicots.

The two orchids contain 115 and 117 R2R3-MYB genes in their genomes, respectively. Feller *et al*.^12^ predicted that the plant R2R3-MYB group underwent extensive amplification before the separation of monocots and dicots but after the separation of plants and animals. This explains why many members of R2R3-MYB genes have been found in orchids. The majority of clades of the R2R3-MYB family are present in both orchids and in the model plant *A*. *thaliana* (Supplementary Fig. 1), suggesting that they were present before the divergence between monocots and dicots. However, there is also an orchid-specific (C17) and a *P*. *equestris*-specific clade (C11), while in both orchids, C3 (S15), C4 (S5) and C13 (S23) are missing (Table 2). It can be deduced that several R2R3-MYB genes might have been lost in orchids during evolution, or experienced an amplification process that might have caused a change in their function in dicots.

### The MYB genes play a role in abiotic stress responses

The MYB genes involved in abiotic stress responses have been widely investigated in plants and have mainly focused on the R2R3-MYB group. For example, R2R3-MYB genes were involved in high temperature stress by increasing the levels of cellular abscisic acid^60^, cold stress by regulating *CBF* genes or ascorbic acid synthesis^56,61^, drought stress^62,63^, and salt stress by regulating ABA signaling^64,65^. Among the 117 R2R3-MYB *D*. *officinale* genes, only nine that were modulated by low temperature were found in this study (Fig. 3). *DoMYB28*, −*29*, −*54*, −*75*, −*78*, −*81*, and −*111* were up-regulated under salinity stress (Fig. 6). The *DoMYB74* and *DoMYB75* homologues, which were recognized as the C2 (S6) clade of R2R3-MYB genes, displayed different expression patterns in response to the two osmotic stresses, PEG and mannitol (Fig. 6). This may due to different *cis*-responsive elements among their transcriptional regulatory regions. For example, the putative promoter of *DoMYB75* contained two ABA response elements, three dehydration response elements, one drought response element, three ethylene response elements, and six MeJA response elements, while *DoMYB74* had only one ABA response element, one dehydration response element, two ethylene response elements, and four MeJA response elements, but no drought response element in its putative promoter (Supplementary Table 3).

MYBR genes are also involved in stress responses. For example, the MYB-related gene *AQUILO* improves cold tolerance in transgenic *A*. *thaliana* and caused the accumulation of oligosaccharides^66^, *OsMYB48*-1 is involved in salt stress by regulating the expression of stress-related genes^67^, and *OsMYBR*1 promoted drought stress in transgenic rice by increasing the free proline and soluble sugar content and up-regulated the expression of stress-related genes under drought treatment^68^. In this work, nine *DoMYBR* genes were up-regulated by cold stress, similar to the above studies, suggesting that the MYB genes in the MYBR or R2R3-MYB groups play roles in plant abiotic stress responses.

### The involvement of R2R3-MYB genes in polysaccharide biosynthesis

Secondary cell walls (SCWs), which are mainly found in plant stems, are primarily composed of cellulose, lignin and hemicelluloses (xylan and glucomannan)^20^. Several TFs are involved in the regulation of SCW biosynthesis. MYB TFs make up the vast majority of TFs in transcriptional regulation of SCW biosynthesis^21^. The R2R3-MYB genes involved in plant SCW biosynthesis are thought to regulate the biosynthesis of SCW polysaccharides. For example, AtMYB46 acts as a regulator in SCW formation and directly regulates the expression of *CSLA9*, which encodes mannan synthase in *A*. *thaliana*^22,69^. *AtMYB7*5 in the C2 (S6) group acts as a regulator in cell wall thickening, testa, as well as biosynthesis of anthocyanins^70–72^. Another MYB gene, *AtMYB113*, in the C2 (S6) subgroup increases pigment production and results in strongly visible anthocyanin pigmentation^73^. *35S::DoMYB75* transgenic Arabidopsis lines showed no anthocyanin, possibly due to the low levels of sucrose present in the medium, but increased WSPs in seeds of transgenic lines (Fig. 7).

In conclusion, 159 and 165 MYB genes were identified from *P*. *equestris* and *D*. *officinale* genomes, respectively. They could be classified into four groups in both orchids: MYBR, R2R3-MYB, 3R-MYB and atypical MYB proteins. Only three R2R3-MYB genes and 12 MYBR genes from *D*. *officinale* were up-regulated under low temperature, suggesting that MYB genes may play a role in the cold stress response in this orchid. Ten R2R3-MYB genes with an expression pattern corresponding to WSP accumulation were identified and regarded as the candidate genes involved in WSP biosynthesis. Over-expression of one candidate gene (*DoMYB75*) in *A*. *thaliana* caused the accumulation of WSPs in *A*. *thaliana* seeds.

## Supplementary information

Supplementary Materials

## Data Availability

The datasets used during the current study are available from the corresponding author on reasonable request.
