# Supplementary material for: Mining MYB transcription factors from the genomes of orchids (Phalaenopsis and Dendrobium) and characterization of an orchid R2R3-MYB gene involved in water-soluble polysaccharide biosynthesis
Source: Sci Rep. 2019 Sep 25;9:13818. doi: 10.1038/s41598-019-49812-8 (PMC6761160; doi:10.1038/s41598-019-49812-8)
Supplement: Supplementary file 1 — Supplementary Materials [file 41598_2019_49812_MOESM1_ESM.pdf]

Mining MYB transcription factors from the genomes of orchids (*Phalaenopsis* and *Dendrobium*) and characterization of an orchid R2R3-MYB gene involved in water-soluble polysaccharide biosynthesis

Chunmei He[1] · Jaime A. Teixeira da Silva[2] · Haobin Wang[1, 3] · Can Si [1, 3] · Mingze Zhang[1,3] · Xiaoming Zhang[1] · Mingzhi Li[4] · Jianwen Tan[5] · Jun Duan[1*]

[1] *Key Laboratory of South China Agricultural Plant Molecular Analysis and Gene Improvement, South China Botanical Garden, Chinese Academy of Sciences, Guangzhou, 510650, China*
[2] *P. O. Box 7, Miki-cho post office, Ikenobe 3011-2, Miki-cho, Kita-gun, Kagawa-ken, 761-0799, Japan*
[3] *University of the Chinese Academy of Sciences, Beijing, 100049, China*
[4] *Biodata Biotechnology Co. Ltd, Heifei, 230031, China*
[5] *College of Forestry and Landscape Architecture, South China Agricultural University, Guangzhou 510642, China.*

[*] Corresponding author: Tel: +86-20-37252993; fax: +86-20-37252978

E-mail address: duanj@scib.ac.cn (Duan J.)

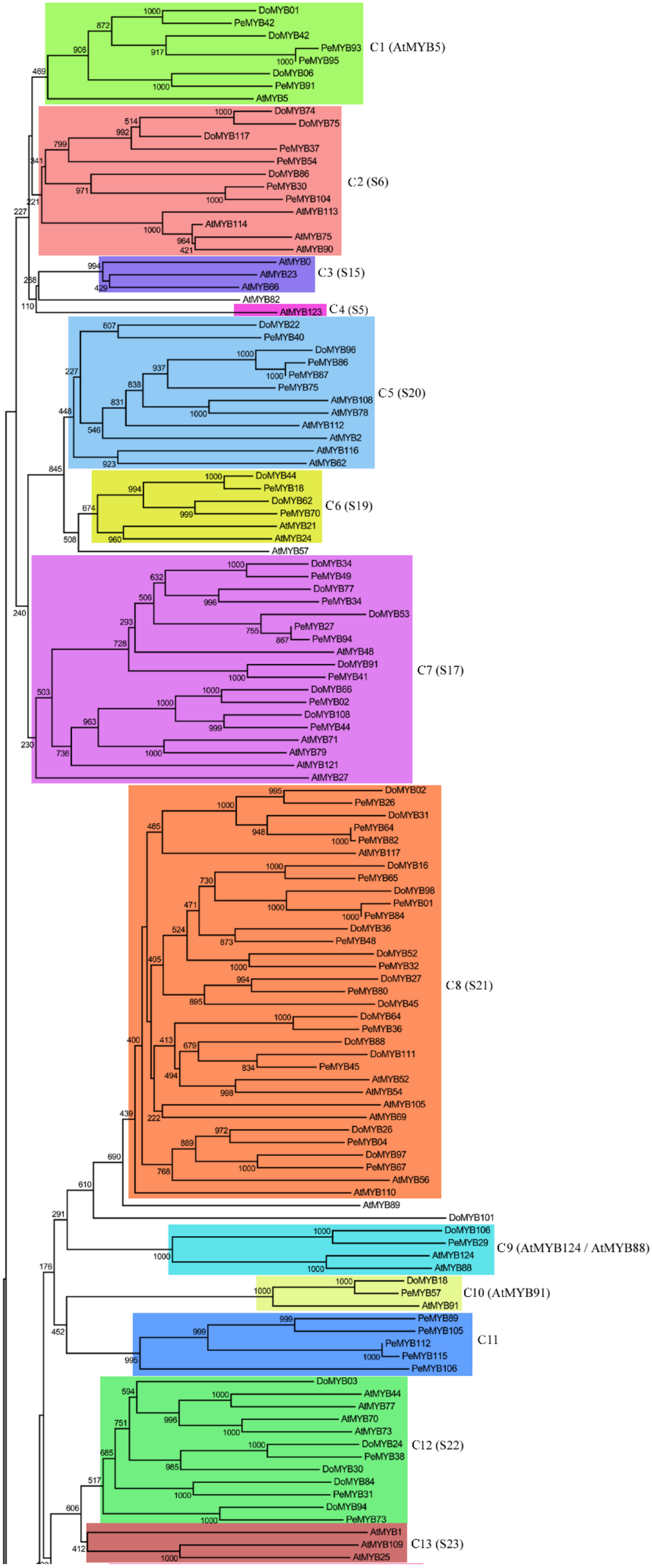
DoMYB01
PeMYB42
DoMYB42
PeMYB93
PeMYB95
DoMYB06
PeMYB91
AtMYB5
C1 (AtMYB5)
DoMYB74
DoMYB75
DoMYB117
PeMYB37
PeMYB54
DoMYB86
PeMYB30
PeMYB104
AtMYB113
AtMYB114
AtMYB75
AtMYB90
C2 (S6)
AtMYB0
AtMYB23
AtMYB66
C3 (S15)
AtMYB82
AtMYB123
C4 (S5)
DoMYB22
PeMYB40
DoMYB96
PeMYB86
PeMYB87
PeMYB75
AtMYB108
AtMYB78
AtMYB112
AtMYB2
AtMYB116
AtMYB62
C5 (S20)
DoMYB44
PeMYB18
DoMYB62
PeMYB70
AtMYB21
AtMYB24
C6 (S19)
AtMYB57
DoMYB34
PeMYB49
DoMYB77
PeMYB34
DoMYB53
PeMYB27
PeMYB94
AtMYB48
DoMYB91
PeMYB41
DoMYB66
PeMYB02
DoMYB108
PeMYB44
AtMYB71
AtMYB79
AtMYB121
AtMYB27
C7 (S17)
DoMYB02
PeMYB26
DoMYB31
PeMYB64
PeMYB82
AtMYB117
DoMYB16
PeMYB65
DoMYB98
PeMYB01
PeMYB84
DoMYB36
PeMYB48
DoMYB52
PeMYB32
DoMYB27
PeMYB80
DoMYB45
DoMYB64
PeMYB36
DoMYB88
DoMYB111
PeMYB45
AtMYB52
AtMYB54
AtMYB105
AtMYB69
DoMYB26
PeMYB04
DoMYB97
PeMYB67
AtMYB56
AtMYB110
C8 (S21)
AtMYB89
DoMYB101
DoMYB106
PeMYB29
AtMYB124
AtMYB88
C9 (AtMYB124 / AtMYB88)
DoMYB18
PeMYB57
AtMYB91
C10 (AtMYB91)
PeMYB89
PeMYB105
PeMYB112
PeMYB115
PeMYB106
C11
DoMYB03
AtMYB44
AtMYB77
AtMYB70
AtMYB73
DoMYB24
PeMYB38
DoMYB30
DoMYB84
PeMYB31
DoMYB94
PeMYB73
C12 (S22)
AtMYB1
AtMYB109
AtMYB25
C13 (S23)

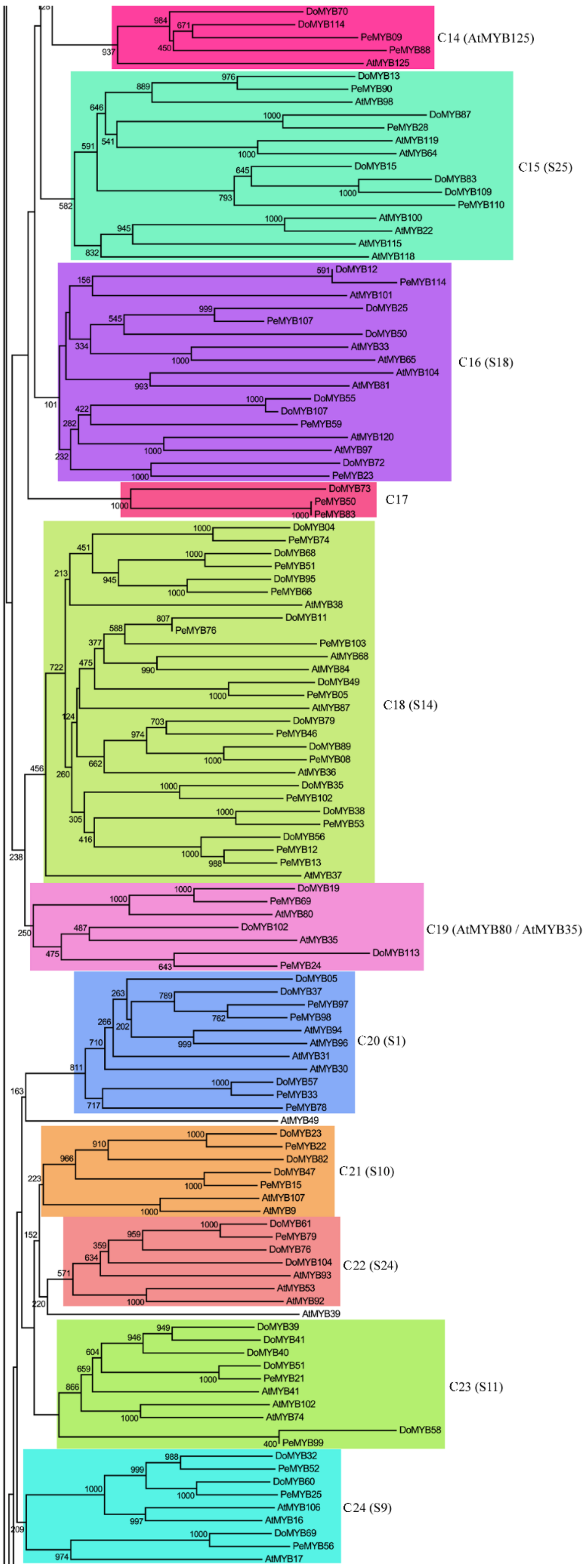
DoMYB70
DoMYB114
PeMYB09
PeMYB88
AtMYB125
C14 (AtMYB125)
DoMYB13
PeMYB90
AtMYB98
DoMYB87
PeMYB28
AtMYB119
AtMYB64
DoMYB15
DoMYB83
DoMYB109
PeMYB110
AtMYB100
AtMYB22
AtMYB115
AtMYB118
C15 (S25)
DoMYB12
PeMYB114
AtMYB101
DoMYB25
PeMYB107
DoMYB50
AtMYB33
AtMYB65
AtMYB104
AtMYB81
DoMYB55
DoMYB107
PeMYB59
AtMYB120
AtMYB97
DoMYB72
PeMYB23
C16 (S18)
DoMYB73
PeMYB50
PeMYB83
C17
DoMYB04
PeMYB74
DoMYB68
PeMYB51
DoMYB95
PeMYB66
AtMYB38
DoMYB11
PeMYB76
PeMYB103
AtMYB68
AtMYB84
DoMYB49
PeMYB05
AtMYB87
DoMYB79
PeMYB46
DoMYB89
PeMYB08
AtMYB36
DoMYB35
PeMYB102
DoMYB38
PeMYB53
DoMYB56
PeMYB12
PeMYB13
AtMYB37
C18 (S14)
DoMYB19
PeMYB69
AtMYB80
DoMYB102
AtMYB35
DoMYB113
PeMYB24
C19 (AtMYB80 / AtMYB35)
DoMYB05
DoMYB37
PeMYB97
PeMYB98
AtMYB94
AtMYB96
AtMYB31
AtMYB30
DoMYB57
PeMYB33
PeMYB78
C20 (S1)
AtMYB49
DoMYB23
PeMYB22
DoMYB82
DoMYB47
PeMYB15
AtMYB107
AtMYB9
C21 (S10)
DoMYB61
PeMYB79
DoMYB76
DoMYB104
AtMYB93
AtMYB53
AtMYB92
C22 (S24)
AtMYB39
DoMYB39
DoMYB41
DoMYB40
DoMYB51
PeMYB21
AtMYB41
AtMYB102
AtMYB74
DoMYB58
PeMYB99
C23 (S11)
DoMYB32
PeMYB52
DoMYB60
PeMYB25
AtMYB106
AtMYB16
DoMYB69
PeMYB56
AtMYB17
C24 (S9)

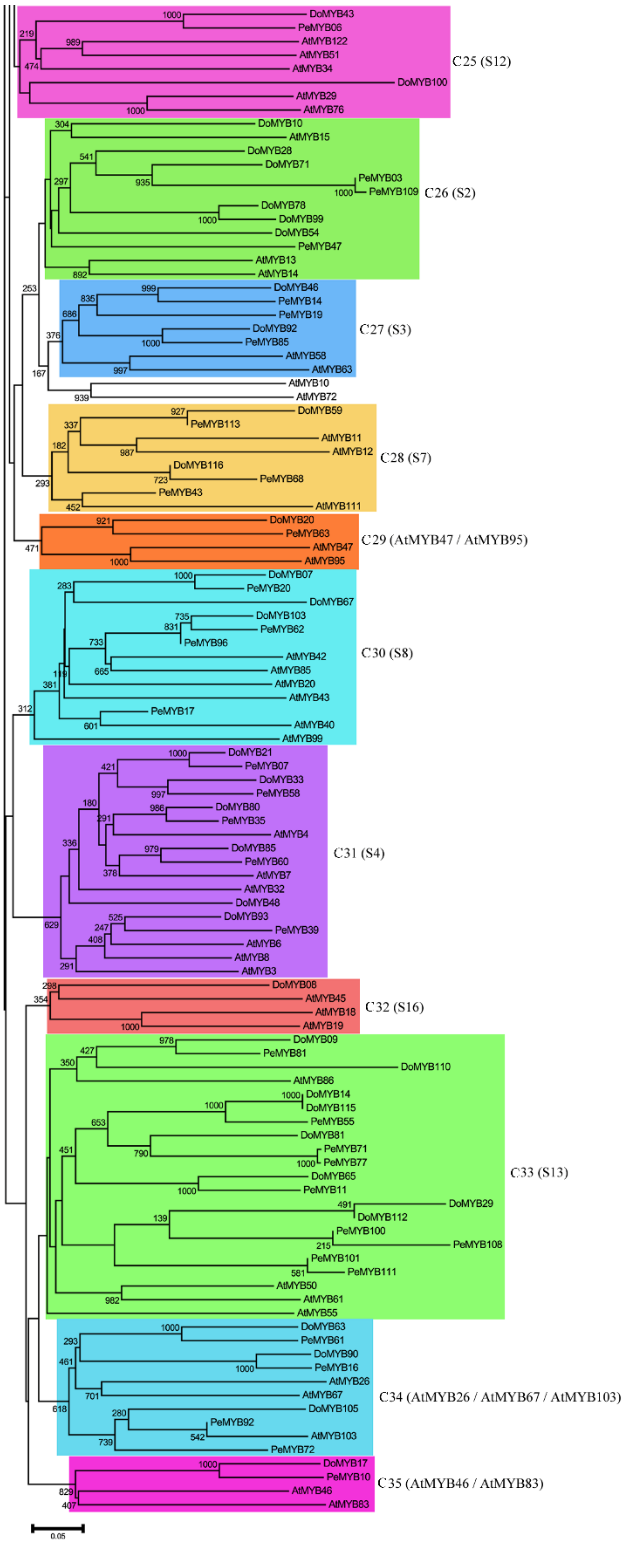

Supplementary Fig. 1 Classification of the total of 2R-MYB proteins from *Arabidopsis thaliana* (At), *P. equestris* (Pe) and *D. officinale* (Do) based on a phylogenetic analysis. The phylogenetic tree was constructed using the Neighbor–Joining method and 1,000 bootstraps with full-length MYB amino acid sequences with ClustalX alignments. The numbers indicated bootstrap support values.

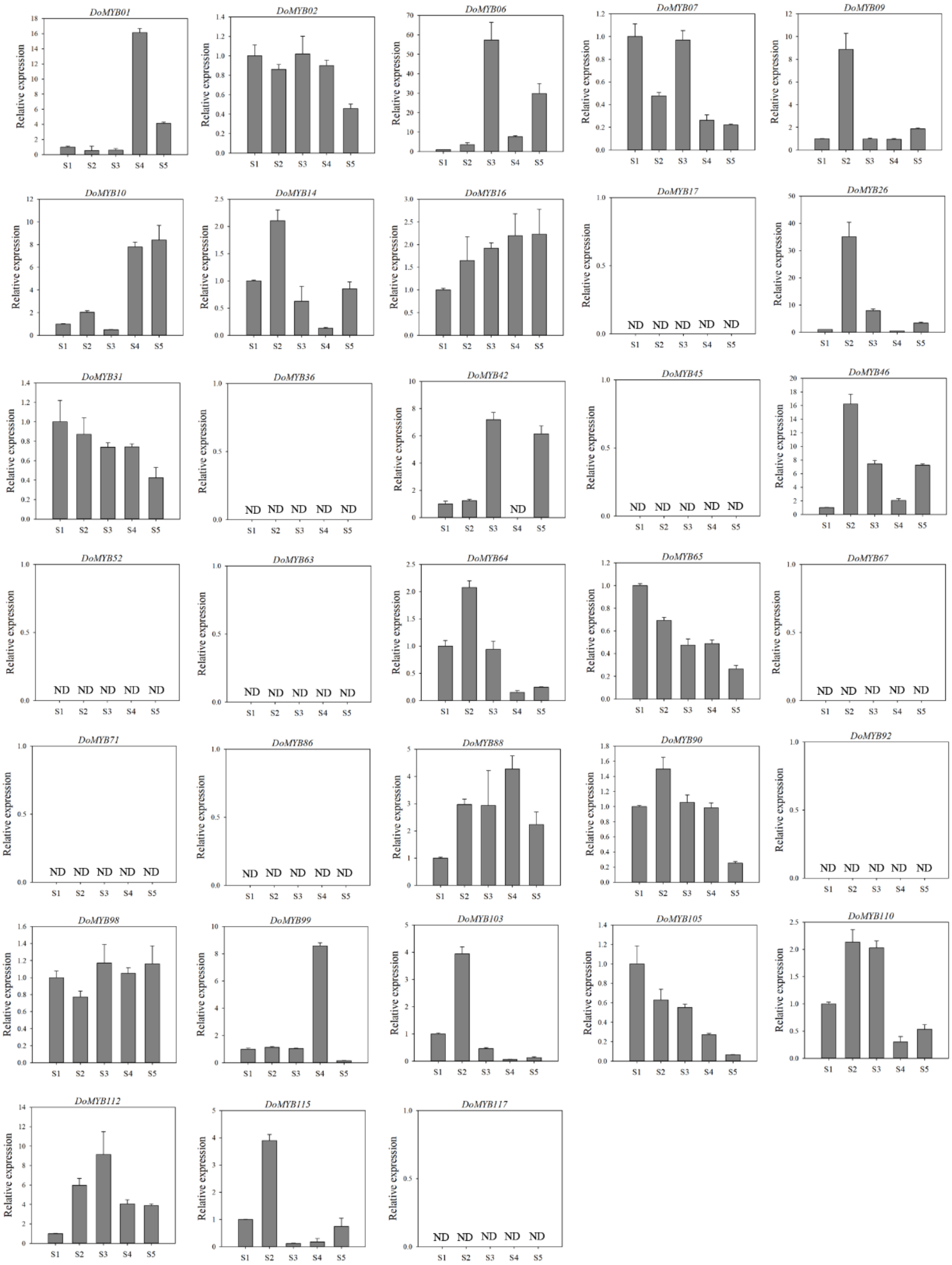

Supplementary Fig. 2 The expression pattern of the remaining genes from the eight clades [C1 (AtMYB5), C2 (S6), C8 (S21), C26 (S2), C27 (S3), C30 (S8), C33 (S13), C34 (AtMYB26/AtMYB67/AtMYB103) and C35 (AtMYB46/AtMYB83)], excluding the 10 genes (*DoMYB27*, *-28*, *-29*, *-54*, *-74*, *-75*, *-78*, *-81*, *-97*, and *-111*) in *D*. *officinale*. Details pertaining to S1-S5 can be found in the materials and methods and results sections. Bars represent mean ± SD (n = 3). Three biological replicates were performed. Different letters in each bar are significantly different at $P < 0.05$ (Duncan's multiple range test). ND, not detected.

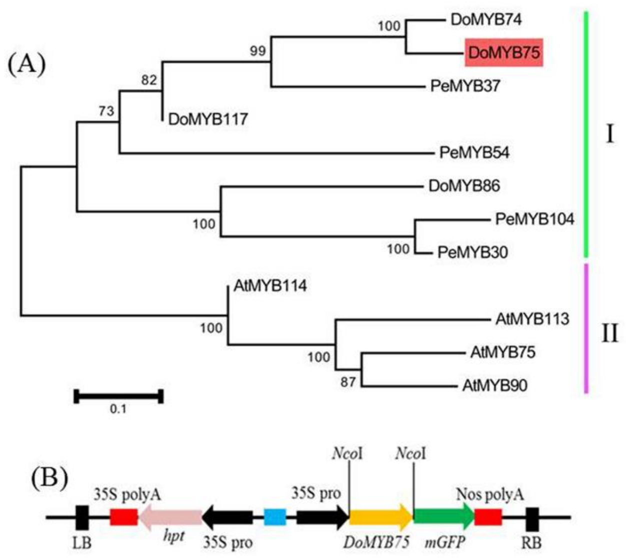

Supplementary Fig. 3 (A). Phylogenetic analysis of C2 (S6) clade proteins from *A. thaliana*, *P. equestris* and *D. officinale*. Phylogenetic tree was generated by MEGA using the Neighbor-joining method with 1000 bootstrap replicates based on MAFFT alignment. (B) Schematic presentation of the *35S*::*DoMYB75* overexpression vector.

Supplementary Table 1 The primers used for qPCR and vector construction.

Primer name	Primer sequence
DoMYB01F	5′-GTTGCAGACTACGATGGATGAA-3′
DoMYB01R	5′-CAGACGGTGAAGGCGAATTA-3′
DoMYB06F	5′-GGAAGAGCTGCAGACTAAGATG-3′
DoMYB06R	5′-CAAAGAATGGAGGCGGATGA-3′
DoMYB42F	5′-GGTGAGAAAGGGACATCAGAAG-3′
DoMYB42R	5′-CCCAATGGGTGGCATTAAGA-3′
DoMYB117F	5′-GGCTCCGATGGCTAAACTATT-3′
DoMYB117R	5′-CCTATTGCCGAGGAGCTTATG-3′
DoMYB74F	5′-AGTTTCAAGGAAGGGCTTAACA-3′
DoMYB74R	5′-AGTCGTCCATTTGCCTTCTC-3′
DoMYB75F	5′-GGCGAGTGTAATGGAGTTTG-3′
DoMYB75R	5′-TCAAATGCAATTGTTAACCTCT-3′
DoMYB86F	5′-GAACTACCTTCGCCCGAATATC-3′
DoMYB86R	5′-GGCAATTAAAGCCCACCTATTG-3′
DoMYB02F	5′-CAATGCTTACAGGAGGAGGAAG-3′
DoMYB02R	5′-CAATGGAGGAAGAGAAGGAGAAG-3′
DoMYB111F	5′-CAGGAAAGAGCTGTAGGTTGAG-3′
DoMYB111R	5′-GAAGCCTCTCTTCTTCCTCTTC-3′
DoMYB16F	5′-ATCACTCCGGCTACTCTGATA-3′
DoMYB16R	5′-GTCGCCCTCTTCAAGGATAAA-3′
DoMYB26F	5′-GGCAACAAATGGGCTCTTATAG-3′
DoMYB26R	5′-TCTGCCTTCTAGCCATGATTAC-3′
DoMYB27F	5′-GGATCGCTTCTTCCACATCTT-3′
DoMYB27R	5′-TCTTCTCTGGTGCTTGTAATGG-3′
DoMYB31F	5′-GTCCTCTCACTCAACTCATCAC-3′
DoMYB31R	5′-TCTCCTCCTCTGCAACATCTA-3′
DoMYB36F	5′-CAGAGAAGCTGGTTGGAAGAT-3′

DoMYB36R	5′-CTTTCTTCCTCCTCCTCAGTAAAG-3′
DoMYB45F	5′-TAGCTGAGAGACAGGGAAGAA-3′
DoMYB45R	5′-CCACGCCTAGGAAATCAAAGA-3′
DoMYB52F	5′-GGAAACAAGTGGGCTCTCATA-3′
DoMYB52R	5′-TGCCTCCTGGCCATAATAAC-3′
DoMYB64F	5′-CTGTGATTGCACGTCTGTTTC-3′
DoMYB64R	5′-CCTTGACCTCTCTCTTACTTTCC-3′
DoMYB88F	5′-GCTAGAAGAGTATGCACCATCTG-3′
DoMYB88R	5′-CCTTCGTCTCCTCCTACGATTA-3′
DoMYB97F	5′-AGCTTGGAGAGTTCGCATTAC-3′
DoMYB97R	5′-CCTGCATATTCCTCATCATCCC-3′
DoMYB98F	5′-GAGACGAACCCTTGTGTATCAG-3′
DoMYB98R	5′-CCATCTGGCTCAACGAGATAAT-3′
DoMYB10F	5′-TGAGCAGTTCCCAGATGTTATG-3′
DoMYB10R	5′-CCCACAAGTCAATCTCACTCTC-3′
DoMYB28F	5′-GCTGCCTGGAAGAACAGATAA-3′
DoMYB28R	5′-CTTCTGCACCTTTGGCTTTG-3′
DoMYB54F	5′-ATCCAAGAGGAAGATGCAAAG-3′A
DoMYB54R	5′-CAGTGCATGAGGAGGAGAAAT-3′
DoMYB71F	5′-TAAGTTGCCGGGAAGAACTG-3′
DoMYB71R	5′-GGACTCCTTGATGGCTTGATTA-3′
DoMYB78F	5′-AGACTTCGGTGGACGAATTATC-3′
DoMYB78R	5′-CAATTGCAGACCATCTGTTTCC-3′
DoMYB99F	5′-GACTTCGGTGGACGAATTATCT-3′
DoMYB99R	5′-TTGCAGCCCATCCATATCC-3′
DoMYB46F	5′-AGGAGAGGGAGGAGTTGATTAG-3′
DoMYB46R	5′-GTAAGAGAGGGTGAGGAGGAG-3′
DoMYB92F	5′-CGGAAGAACAGACAACGAGATA-3′
DoMYB92R	5′-TGTTGGAGAAGACGAGGATTG-3′

DoMYB07F	5′-ATTGACCCTCTCACACACAAG-3′
DoMYB07R	5′-CGCAGTTGATAAGTCGGAGTAG-3′
DoMYB103F	5′-GAGAGTACCAACCCACAACAA-3′
DoMYB103R	5′-CTCCTCTCCCAAGCTTCATTAC-3′
DoMYB67F	5′-CAGGTTTGATGAGATGTGGTAAGA-3′
DoMYB67R	5′-GCTGCTCCTCAGTTTCAGATAG-3′
DoMYB09F	5′-CTGAAGCAGAAGCTGAGGAA-3′
DoMYB09R	5′-CCAGCAACCAACACCAAATC-3′
DoMYB110F	5′-CTTCGCCGCTCTAAACAAATC-3′
DoMYB110R	5′-CTTCGTCAGGAGACCATAATCC-3′
DoMYB112F	5′-CCCTTGGACACTTGAAGAAGA-3′
DoMYB112R	5′-GAGGAAGAATACAATACCAACAAGC -3′
DoMYB115F	5′-CTGCAGGCTTAGGTGGATAAA-3′
DoMYB115R	5′-CTGAGACCATCTGTTTCCAAGA-3′
DoMYB14F	5′-CTGCAGGCTTAGGTGGATAAA-3′
DoMYB14R	5′-CTGAGACCATCTGTTTCCAAGA-3′
DoMYB29F	5′-CTCTTCTAGAGCCAGCCATTATC-3′
DoMYB29R	5′-CAAGTGGAGGGAGGTCAAATAA-3′
DoMYB65F	5′-GCTGCGGTGGATTAACTATCT-3′
DoMYB65R	5′-CTGAGACCACCTGTTTCCTAAA-3′
DoMYB81F	5′-GGGACAGTGAAGAGATGGAAAG-3′
DoMYB81R	5′-GGAGGTCTCCAGCATGTAAAG-3′
DoMYB105F	5′-GGAAACCGATGGGCTCATATT-3′
DoMYB105R	5′-CCTGTGTTCGGCTTTCTTATCT-3′
DoMYB63F	5′-GGAAACAGATGGGCACAGATAG-3′
DoMYB63R	5′-TGAGAGATTAGGAAGCGAGAGG-3′
DoMYB90F	5′-GTCTCCAGAGGAAGATGAGAAG-3′
DoMYB90R	5′-CTGCAACCCTGCTTGTTTAG-3′
DoMYB17F	5′-GCCAGGTCGCACAGATAAT-3′

DoMYB17R	5′-TTGGTCATGAGTTGAGGAAGATAG-3′
OxDoMYB75F	5′-GGACTCTTGACCATGGCGATGGGAAGGAATTCGT GC-3′
OxDoMYB75R	5′-GTCAGATCTACCATGGTGAATCCACCTCCAATATCAA-3′
AtUBQ10F	5'-GATCTTTGCCGGAAAACAATTGGAGGATGGT-3'
AtUBQ10R	5'-CGACTTGTCATTAGAAAGAAAGAGATAACAGG-3'

Supplementary Table 2 Information of MYB family genes in the tested two orchid species.

Subfamily	Gene name	Locus name	Species
1RMYB	DoMYBR01	Dof000365	*Dendrobium officinale*
1RMYB	DoMYBR02	Dof000742	*Dendrobium officinale*
1RMYB	DoMYBR03	Dof002404	*Dendrobium officinale*
1RMYB	DoMYBR04	Dof003868	*Dendrobium officinale*
1RMYB	DoMYBR05	Dof005654	*Dendrobium officinale*
1RMYB	DoMYBR06	Dof005716	*Dendrobium officinale*
1RMYB	DoMYBR07	Dof006201	*Dendrobium officinale*
1RMYB	DoMYBR08	Dof006450	*Dendrobium officinale*
1RMYB	DoMYBR09	Dof006907	*Dendrobium officinale*
1RMYB	DoMYBR10	Dof007033	*Dendrobium officinale*
1RMYB	DoMYBR11	Dof007991	*Dendrobium officinale*
1RMYB	DoMYBR12	Dof008709	*Dendrobium officinale*
1RMYB	DoMYBR13	Dof009011	*Dendrobium officinale*
1RMYB	DoMYBR14	Dof009357	*Dendrobium officinale*
1RMYB	DoMYBR15	Dof009473	*Dendrobium officinale*
1RMYB	DoMYBR16	Dof009580	*Dendrobium officinale*
1RMYB	DoMYBR17	Dof011133	*Dendrobium officinale*
1RMYB	DoMYBR18	Dof013211	*Dendrobium officinale*
1RMYB	DoMYBR19	Dof013359	*Dendrobium officinale*
1RMYB	DoMYBR20	Dof013785	*Dendrobium officinale*
1RMYB	DoMYBR21	Dof014135	*Dendrobium officinale*
1RMYB	DoMYBR22	Dof014999	*Dendrobium officinale*
1RMYB	DoMYBR23	Dof015140	*Dendrobium officinale*
1RMYB	DoMYBR24	Dof015718	*Dendrobium officinale*
1RMYB	DoMYBR25	Dof016165	*Dendrobium officinale*
1RMYB	DoMYBR26	Dof016566	*Dendrobium officinale*
1RMYB	DoMYBR27	Dof017492	*Dendrobium officinale*
1RMYB	DoMYBR28	Dof017942	*Dendrobium officinale*
1RMYB	DoMYBR29	Dof018315	*Dendrobium officinale*
1RMYB	DoMYBR30	Dof018489	*Dendrobium officinale*
1RMYB	DoMYBR31	Dof019422	*Dendrobium officinale*
1RMYB	DoMYBR32	Dof021196	*Dendrobium officinale*
1RMYB	DoMYBR33	Dof021970	*Dendrobium officinale*
1RMYB	DoMYBR34	Dof022433	*Dendrobium officinale*
1RMYB	DoMYBR35	Dof023758	*Dendrobium officinale*
1RMYB	DoMYBR36	Dof024354	*Dendrobium officinale*
1RMYB	DoMYBR37	Dof024428	*Dendrobium officinale*
1RMYB	DoMYBR38	Dof025998	*Dendrobium officinale*
1RMYB	DoMYBR39	Dof026974	*Dendrobium officinale*
1RMYB	DoMYBR40	Dof027374	*Dendrobium officinale*
1RMYB	DoMYBR41	Dof027884	*Dendrobium officinale*
1RMYB	DoMYBR42	Dof028360	*Dendrobium officinale*

2RMYB	DoMYB01	Dof000330	*Dendrobium officinale*
2RMYB	DoMYB07	Dof002120	*Dendrobium officinale*
2RMYB	DoMYB17	Dof003857	*Dendrobium officinale*
2RMYB	DoMYB18	Dof003883	*Dendrobium officinale*
2RMYB	DoMYB19	Dof004076	*Dendrobium officinale*
2RMYB	DoMYB34	Dof006651	*Dendrobium officinale*
2RMYB	DoMYB63	Dof013354	*Dendrobium officinale*
2RMYB	DoMYB66	Dof014058	*Dendrobium officinale*
2RMYB	DoMYB70	Dof015141	*Dendrobium officinale*
2RMYB	DoMYB77	Dof016720	*Dendrobium officinale*
2RMYB	DoMYB86	Dof019793	*Dendrobium officinale*
2RMYB	DoMYB90	Dof020577	*Dendrobium officinale*
2RMYB	DoMYB91	Dof021348	*Dendrobium officinale*
2RMYB	DoMYB103	Dof024763	*Dendrobium officinale*
2RMYB	DoMYB108	Dof025989	*Dendrobium officinale*
2RMYB	DoMYB114	Dof027670	*Dendrobium officinale*
2RMYB	DoMYB05	Dof001786	*Dendrobium officinale*
2RMYB	DoMYB37	Dof007212	*Dendrobium officinale*
2RMYB	DoMYB57	Dof011908	*Dendrobium officinale*
2RMYB	DoMYB23	Dof004449	*Dendrobium officinale*
2RMYB	DoMYB47	Dof008800	*Dendrobium officinale*
2RMYB	DoMYB82	Dof018074	*Dendrobium officinale*
2RMYB	DoMYB39	Dof007631	*Dendrobium officinale*
2RMYB	DoMYB40	Dof007632	*Dendrobium officinale*
2RMYB	DoMYB41	Dof007634	*Dendrobium officinale*
2RMYB	DoMYB51	Dof010446	*Dendrobium officinale*
2RMYB	DoMYB14	Dof003055	*Dendrobium officinale*
2RMYB	DoMYB65	Dof013578	*Dendrobium officinale*
2RMYB	DoMYB115	Dof027693	*Dendrobium officinale*
2RMYB	DoMYB04	Dof001651	*Dendrobium officinale*
2RMYB	DoMYB11	Dof002872	*Dendrobium officinale*
2RMYB	DoMYB35	Dof006663	*Dendrobium officinale*
2RMYB	DoMYB49	Dof009302	*Dendrobium officinale*
2RMYB	DoMYB56	Dof011001	*Dendrobium officinale*
2RMYB	DoMYB68	Dof015064	*Dendrobium officinale*
2RMYB	DoMYB79	Dof017102	*Dendrobium officinale*
2RMYB	DoMYB89	Dof020354	*Dendrobium officinale*
2RMYB	DoMYB08	Dof002298	*Dendrobium officinale*
2RMYB	DoMYB43	Dof008095	*Dendrobium officinale*
2RMYB	DoMYB25	Dof004516	*Dendrobium officinale*
2RMYB	DoMYB50	Dof009710	*Dendrobium officinale*
2RMYB	DoMYB55	Dof010971	*Dendrobium officinale*
2RMYB	DoMYB72	Dof015821	*Dendrobium officinale*
2RMYB	DoMYB73	Dof016066	*Dendrobium officinale*

2RMYB	DoMYB44	Dof008253	*Dendrobium officinale*
2RMYB	DoMYB62	Dof013210	*Dendrobium officinale*
2RMYB	DoMYB06	Dof001900	*Dendrobium officinale*
2RMYB	DoMYB10	Dof002831	*Dendrobium officinale*
2RMYB	DoMYB28	Dof005663	*Dendrobium officinale*
2RMYB	DoMYB42	Dof007767	*Dendrobium officinale*
2RMYB	DoMYB54	Dof010873	*Dendrobium officinale*
2RMYB	DoMYB71	Dof015147	*Dendrobium officinale*
2RMYB	DoMYB78	Dof016977	*Dendrobium officinale*
2RMYB	DoMYB99	Dof023028	*Dendrobium officinale*
2RMYB	DoMYB22	Dof004266	*Dendrobium officinale*
2RMYB	DoMYB96	Dof021926	*Dendrobium officinale*
2RMYB	DoMYB02	Dof000975	*Dendrobium officinale*
2RMYB	DoMYB16	Dof003332	*Dendrobium officinale*
2RMYB	DoMYB26	Dof004711	*Dendrobium officinale*
2RMYB	DoMYB27	Dof005061	*Dendrobium officinale*
2RMYB	DoMYB31	Dof006370	*Dendrobium officinale*
2RMYB	DoMYB36	Dof007074	*Dendrobium officinale*
2RMYB	DoMYB45	Dof008464	*Dendrobium officinale*
2RMYB	DoMYB52	Dof010799	*Dendrobium officinale*
2RMYB	DoMYB64	Dof013395	*Dendrobium officinale*
2RMYB	DoMYB88	Dof020290	*Dendrobium officinale*
2RMYB	DoMYB97	Dof022364	*Dendrobium officinale*
2RMYB	DoMYB98	Dof023000	*Dendrobium officinale*
2RMYB	DoMYB03	Dof001371	*Dendrobium officinale*
2RMYB	DoMYB24	Dof004485	*Dendrobium officinale*
2RMYB	DoMYB30	Dof006288	*Dendrobium officinale*
2RMYB	DoMYB84	Dof018580	*Dendrobium officinale*
2RMYB	DoMYB46	Dof008796	*Dendrobium officinale*
2RMYB	DoMYB92	Dof021495	*Dendrobium officinale*
2RMYB	DoMYB21	Dof004179	*Dendrobium officinale*
2RMYB	DoMYB33	Dof006582	*Dendrobium officinale*
2RMYB	DoMYB48	Dof009078	*Dendrobium officinale*
2RMYB	DoMYB75	Dof016103	*Dendrobium officinale*
2RMYB	DoMYB80	Dof017534	*Dendrobium officinale*
2RMYB	DoMYB85	Dof018958	*Dendrobium officinale*
2RMYB	DoMYB93	Dof021503	*Dendrobium officinale*
2RMYB	DoMYB74	Dof016101	*Dendrobium officinale*
2RMYB	DoMYB59	Dof012025	*Dendrobium officinale*
2RMYB	DoMYB32	Dof006378	*Dendrobium officinale*
2RMYB	DoMYB60	Dof012359	*Dendrobium officinale*
2RMYB	DoMYB69	Dof015106	*Dendrobium officinale*
2RMYB	DoMYB107	Dof025601	*Dendrobium officinale*
2RMYB	DoMYB95	Dof021862	*Dendrobium officinale*

2RMYB	DoMYB15	Dof003292	*Dendrobium officinale*
2RMYB	DoMYB13	Dof002914	*Dendrobium officinale*
2RMYB	DoMYB20	Dof004096	*Dendrobium officinale*
2RMYB	DoMYB29	Dof005728	*Dendrobium officinale*
2RMYB	DoMYB53	Dof010823	*Dendrobium officinale*
2RMYB	DoMYB67	Dof014629	*Dendrobium officinale*
2RMYB	DoMYB83	Dof018453	*Dendrobium officinale*
2RMYB	DoMYB87	Dof020126	*Dendrobium officinale*
2RMYB	DoMYB105	Dof025123	*Dendrobium officinale*
2RMYB	DoMYB106	Dof025542	*Dendrobium officinale*
2RMYB	DoMYB109	Dof026498	*Dendrobium officinale*
2RMYB	DoMYB113	Dof027052	*Dendrobium officinale*
2RMYB	DoMYB09	Dof002384	*Dendrobium officinale*
2RMYB	DoMYB110	Dof026606	*Dendrobium officinale*
2RMYB	DoMYB12	Dof002880	*Dendrobium officinale*
2RMYB	DoMYB101	Dof024468	*Dendrobium officinale*
2RMYB	DoMYB111	Dof026832	*Dendrobium officinale*
2RMYB	DoMYB94	Dof021702	*Dendrobium officinale*
2RMYB	DoMYB102	Dof024573	*Dendrobium officinale*
2RMYB	DoMYB104	Dof025065	*Dendrobium officinale*
2RMYB	DoMYB112	Dof026977	*Dendrobium officinale*
2RMYB	DoMYB100	Dof023466	*Dendrobium officinale*
2RMYB	DoMYB117	Dof027975	*Dendrobium officinale*
2RMYB	DoMYB58	Dof011977	*Dendrobium officinale*
2RMYB	DoMYB116	Dof027920	*Dendrobium officinale*
2RMYB	DoMYB61	Dof012767	*Dendrobium officinale*
2RMYB	DoMYB76	Dof016187	*Dendrobium officinale*
2RMYB	DoMYB81	Dof018028	*Dendrobium officinale*
2RMYB	DoMYB38	Dof007558	*Dendrobium officinale*
3RMYB	DoMYB3R1	Dof001314	*Dendrobium officinale*
3RMYB	DoMYB3R2	Dof005624	*Dendrobium officinale*
3RMYB	DoMYB3R3	Dof026134	*Dendrobium officinale*
3RMYB	DoMYB3R4	Dof003882	*Dendrobium officinale*
4RMYB	DoMYB4R1	Dof002676	*Dendrobium officinale*
CDC	DoMYBCDC	Dof000609	*Dendrobium officinale*
1RMYB	PeMYBR01	PEQU_02344	*Phalaenopsis equestris*
1RMYB	PeMYBR02	PEQU_03826	*Phalaenopsis equestris*
1RMYB	PeMYBR03	PEQU_09358	*Phalaenopsis equestris*
1RMYB	PeMYBR04	PEQU_28155	*Phalaenopsis equestris*
1RMYB	PeMYBR05	PEQU_02305	*Phalaenopsis equestris*

1RMYB	PeMYBR06	PEQU_07911	*Phalaenopsis equestris*
1RMYB	PeMYBR07	PEQU_09649	*Phalaenopsis equestris*
1RMYB	PeMYBR08	PEQU_17862	*Phalaenopsis equestris*
1RMYB	PeMYBR09	PEQU_18861	*Phalaenopsis equestris*
1RMYB	PeMYBR10	PEQU_19987	*Phalaenopsis equestris*
1RMYB	PeMYBR11	PEQU_20883	*Phalaenopsis equestris*
1RMYB	PeMYBR12	PEQU_21184	*Phalaenopsis equestris*
1RMYB	PeMYBR13	PEQU_21502	*Phalaenopsis equestris*
1RMYB	PeMYBR14	PEQU_22681	*Phalaenopsis equestris*
1RMYB	PeMYBR15	PEQU_31813	*Phalaenopsis equestris*
1RMYB	PeMYBR16	PEQU_34212	*Phalaenopsis equestris*
1RMYB	PeMYBR17	PEQU_37054	*Phalaenopsis equestris*
1RMYB	PeMYBR18	PEQU_38150	*Phalaenopsis equestris*
1RMYB	PeMYBR19	PEQU_38447	*Phalaenopsis equestris*
1RMYB	PeMYBR20	PEQU_00313	*Phalaenopsis equestris*
1RMYB	PeMYBR21	PEQU_06243	*Phalaenopsis equestris*
1RMYB	PeMYBR22	PEQU_07630	*Phalaenopsis equestris*
1RMYB	PeMYBR23	PEQU_14354	*Phalaenopsis equestris*
1RMYB	PeMYBR24	PEQU_30334	*Phalaenopsis equestris*
1RMYB	PeMYBR25	PEQU_05110	*Phalaenopsis equestris*
1RMYB	PeMYBR26	PEQU_08237	*Phalaenopsis equestris*
1RMYB	PeMYBR27	PEQU_25415	*Phalaenopsis equestris*
1RMYB	PeMYBR28	PEQU_40552	*Phalaenopsis equestris*
1RMYB	PeMYBR29	PEQU_03067	*Phalaenopsis equestris*
1RMYB	PeMYBR30	PEQU_09194	*Phalaenopsis equestris*
1RMYB	PeMYBR31	PEQU_10019	*Phalaenopsis equestris*
1RMYB	PeMYBR32	PEQU_11296	*Phalaenopsis equestris*
1RMYB	PeMYBR33	PEQU_17373	*Phalaenopsis equestris*
1RMYB	PeMYBR34	PEQU_27771	*Phalaenopsis equestris*
1RMYB	PeMYBR35	PEQU_36853	*Phalaenopsis equestris*
1RMYB	PeMYBR36	PEQU_10426	*Phalaenopsis equestris*
1RMYB	PeMYBR37	PEQU_21566	*Phalaenopsis equestris*
1RMYB	PeMYBR38	PEQU_01675	*Phalaenopsis equestris*
1RMYB	PeMYBR39	PEQU_41557	*Phalaenopsis equestris*
1RMYB	PeMYBR40	PEQU_27639	*Phalaenopsis equestris*
2RMYB	PeMYB01	PEQU_00368	*Phalaenopsis equestris*
2RMYB	PeMYB02	PEQU_00728	*Phalaenopsis equestris*
2RMYB	PeMYB04	PEQU_02109	*Phalaenopsis equestris*
2RMYB	PeMYB05	PEQU_02591	*Phalaenopsis equestris*
2RMYB	PeMYB06	PEQU_03213	*Phalaenopsis equestris*
2RMYB	PeMYB07	PEQU_03393	*Phalaenopsis equestris*
2RMYB	PeMYB08	PEQU_04692	*Phalaenopsis equestris*
2RMYB	PeMYB09	PEQU_04858	*Phalaenopsis equestris*
2RMYB	PeMYB10	PEQU_05034	*Phalaenopsis equestris*

2RMYB	PeMYB11	PEQU_05119	*Phalaenopsis equestris*
2RMYB	PeMYB13	PEQU_05683	*Phalaenopsis equestris*
2RMYB	PeMYB14	PEQU_05903	*Phalaenopsis equestris*
2RMYB	PeMYB15	PEQU_05918	*Phalaenopsis equestris*
2RMYB	PeMYB17	PEQU_06390	*Phalaenopsis equestris*
2RMYB	PeMYB18	PEQU_06488	*Phalaenopsis equestris*
2RMYB	PeMYB19	PEQU_06600	*Phalaenopsis equestris*
2RMYB	PeMYB20	PEQU_06922	*Phalaenopsis equestris*
2RMYB	PeMYB21	PEQU_07382	*Phalaenopsis equestris*
2RMYB	PeMYB22	PEQU_07503	*Phalaenopsis equestris*
2RMYB	PeMYB23	PEQU_07637	*Phalaenopsis equestris*
2RMYB	PeMYB24	PEQU_08801	*Phalaenopsis equestris*
2RMYB	PeMYB26	PEQU_09074	*Phalaenopsis equestris*
2RMYB	PeMYB27	PEQU_09152	*Phalaenopsis equestris*
2RMYB	PeMYB29	PEQU_09503	*Phalaenopsis equestris*
2RMYB	PeMYB30	PEQU_10362	*Phalaenopsis equestris*
2RMYB	PeMYB31	PEQU_10550	*Phalaenopsis equestris*
2RMYB	PeMYB32	PEQU_10664	*Phalaenopsis equestris*
2RMYB	PeMYB33	PEQU_10683	*Phalaenopsis equestris*
2RMYB	PeMYB34	PEQU_10762	*Phalaenopsis equestris*
2RMYB	PeMYB35	PEQU_10866	*Phalaenopsis equestris*
2RMYB	PeMYB36	PEQU_10971	*Phalaenopsis equestris*
2RMYB	PeMYB38	PEQU_12592	*Phalaenopsis equestris*
2RMYB	PeMYB39	PEQU_12801	*Phalaenopsis equestris*
2RMYB	PeMYB40	PEQU_13544	*Phalaenopsis equestris*
2RMYB	PeMYB41	PEQU_14300	*Phalaenopsis equestris*
2RMYB	PeMYB42	PEQU_14547	*Phalaenopsis equestris*
2RMYB	PeMYB43	PEQU_14880	*Phalaenopsis equestris*
2RMYB	PeMYB44	PEQU_15899	*Phalaenopsis equestris*
2RMYB	PeMYB45	PEQU_16171	*Phalaenopsis equestris*
2RMYB	PeMYB46	PEQU_16389	*Phalaenopsis equestris*
2RMYB	PeMYB47	PEQU_17021	*Phalaenopsis equestris*
2RMYB	PeMYB48	PEQU_17178	*Phalaenopsis equestris*
2RMYB	PeMYB49	PEQU_17258	*Phalaenopsis equestris*
2RMYB	PeMYB50	PEQU_18431	*Phalaenopsis equestris*
2RMYB	PeMYB51	PEQU_18783	*Phalaenopsis equestris*
2RMYB	PeMYB52	PEQU_19266	*Phalaenopsis equestris*
2RMYB	PeMYB54	PEQU_20333	*Phalaenopsis equestris*
2RMYB	PeMYB55	PEQU_20514	*Phalaenopsis equestris*
2RMYB	PeMYB56	PEQU_21248	*Phalaenopsis equestris*
2RMYB	PeMYB57	PEQU_21694	*Phalaenopsis equestris*
2RMYB	PeMYB58	PEQU_23041	*Phalaenopsis equestris*
2RMYB	PeMYB59	PEQU_23598	*Phalaenopsis equestris*
2RMYB	PeMYB60	PEQU_23940	*Phalaenopsis equestris*

2RMYB	PeMYB61	PEQU_24421	*Phalaenopsis equestris*
2RMYB	PeMYB62	PEQU_25920	*Phalaenopsis equestris*
2RMYB	PeMYB65	PEQU_29143	*Phalaenopsis equestris*
2RMYB	PeMYB66	PEQU_29475	*Phalaenopsis equestris*
2RMYB	PeMYB67	PEQU_29826	*Phalaenopsis equestris*
2RMYB	PeMYB68	PEQU_30611	*Phalaenopsis equestris*
2RMYB	PeMYB71	PEQU_31568	*Phalaenopsis equestris*
2RMYB	PeMYB72	PEQU_31880	*Phalaenopsis equestris*
2RMYB	PeMYB73	PEQU_32696	*Phalaenopsis equestris*
2RMYB	PeMYB74	PEQU_32963	*Phalaenopsis equestris*
2RMYB	PeMYB75	PEQU_33555	*Phalaenopsis equestris*
2RMYB	PeMYB76	PEQU_33827	*Phalaenopsis equestris*
2RMYB	PeMYB77	PEQU_34202	*Phalaenopsis equestris*
2RMYB	PeMYB78	PEQU_34697	*Phalaenopsis equestris*
2RMYB	PeMYB79	PEQU_36816	*Phalaenopsis equestris*
2RMYB	PeMYB80	PEQU_36904	*Phalaenopsis equestris*
2RMYB	PeMYB81	PEQU_39789	*Phalaenopsis equestris*
2RMYB	PeMYB82	PEQU_40332	*Phalaenopsis equestris*
2RMYB	PeMYB83	PEQU_40608	*Phalaenopsis equestris*
2RMYB	PeMYB84	PEQU_40802	*Phalaenopsis equestris*
2RMYB	PeMYB86	PEQU_42161	*Phalaenopsis equestris*
2RMYB	PeMYB87	PEQU_29058	*Phalaenopsis equestris*
2RMYB	PeMYB88	PEQU_01676	*Phalaenopsis equestris*
2RMYB	PeMYB89	PEQU_05560	*Phalaenopsis equestris*
2RMYB	PeMYB90	PEQU_08721	*Phalaenopsis equestris*
2RMYB	PeMYB91	PEQU_16344	*Phalaenopsis equestris*
2RMYB	PeMYB92	PEQU_39245	*Phalaenopsis equestris*
2RMYB	PeMYB93	PEQU_39447	*Phalaenopsis equestris*
2RMYB	PeMYB94	PEQU_39933	*Phalaenopsis equestris*
2RMYB	PeMYB95	PEQU_40997	*Phalaenopsis equestris*
2RMYB	PeMYB96	PEQU_41616	*Phalaenopsis equestris*
2RMYB	PeMYB97	PEQU_03031	*Phalaenopsis equestris*
2RMYB	PeMYB98	PEQU_10878	*Phalaenopsis equestris*
2RMYB	PeMYB99	PEQU_42224	*Phalaenopsis equestris*
2RMYB	PeMYB100	PEQU_38668	*Phalaenopsis equestris*
2RMYB	PeMYB101	PEQU_39588	*Phalaenopsis equestris*
2RMYB	PeMYB102	PEQU_37625	*Phalaenopsis equestris*
2RMYB	PeMYB103	PEQU_41790	*Phalaenopsis equestris*
2RMYB	PeMYB104	PEQU_10361	*Phalaenopsis equestris*
2RMYB	PeMYB105	PEQU_05558	*Phalaenopsis equestris*
2RMYB	PeMYB106	PEQU_05554	*Phalaenopsis equestris*
2RMYB	PeMYB107	PEQU_09423	*Phalaenopsis equestris*
2RMYB	PeMYB108	PEQU_36948	*Phalaenopsis equestris*
2RMYB	PeMYB109	PEQU_40030	*Phalaenopsis equestris*

2RMYB	PeMYB110	PEQU_31816	*Phalaenopsis equestris*
2RMYB	PeMYB111	PEQU_29587	*Phalaenopsis equestris*
2RMYB	PeMYB112	PEQU_05556	*Phalaenopsis equestris*
2RMYB	PeMYB113	PEQU_34075	*Phalaenopsis equestris*
2RMYB	PeMYB114	PEQU_41442	*Phalaenopsis equestris*
2RMYB	PeMYB115	PEQU_22788	*Phalaenopsis equestris*
2RMYB	PeMYB28	PEQU_09363	*Phalaenopsis equestris*
2RMYB	PeMYB03	PEQU_01880	*Phalaenopsis equestris*
2RMYB	PeMYB64	PEQU_28311	*Phalaenopsis equestris*
2RMYB	PeMYB12	PEQU_05643	*Phalaenopsis equestris*
2RMYB	PeMYB16	PEQU_06231	*Phalaenopsis equestris*
2RMYB	PeMYB25	PEQU_09064	*Phalaenopsis equestris*
2RMYB	PeMYB37	PEQU_11292	*Phalaenopsis equestris*
2RMYB	PeMYB53	PEQU_19346	*Phalaenopsis equestris*
2RMYB	PeMYB63	PEQU_27252	*Phalaenopsis equestris*
2RMYB	PeMYB69	PEQU_30955	*Phalaenopsis equestris*
2RMYB	PeMYB70	PEQU_31459	*Phalaenopsis equestris*
2RMYB	PeMYB85	PEQU_41555	*Phalaenopsis equestris*
3RMYB	PeMYB3R1	PEQU_09277	*Phalaenopsis equestris*
3RMYB	PeMYB3R2	PEQU_13832	*Phalaenopsis equestris*
3RMYB	PeMYB3R3	PEQU_05555	*Phalaenopsis equestris*
CDC	PeMYBCDC	PEQU_33908	*Phalaenopsis equestris*

Supplementary Table 3 The *cis*-responsive elements of MYB genes from *P. equestris* and *D. officinale*

	A	B	C	D	E	F	G	H	I	J	K	L	M	N	O	P	Q	R	S	T	U
DoCDC	7	0	0	1	0	0	0	0	3	0	0	30	2	4	1	0	0	1	0	3	1
DoMYB01	2	1	0	1	0	0	0	0	4	1	0	7	1	0	0	0	0	3	0	7	1
DoMYB02	4	3	0	0	1	0	0	0	3	3	0	13	2	0	0	0	0	1	0	3	1
DoMYB03	8	1	0	0	0	0	0	1	7	2	0	10	14	0	0	0	0	1	0	0	11
DoMYB04	2	2	0	0	0	0	1	1	2	0	0	9	5	2	1	0	0	1	1	3	12
DoMYB05	0	0	0	0	0	0	0	0	0	0	0	0	1	0	0	0	0	0	0	0	0
DoMYB06	8	1	0	0	1	0	0	1	2	4	0	12	1	4	3	0	0	1	0	0	2
DoMYB07	11	1	0	1	0	0	0	0	0	1	0	10	1	2	1	0	0	4	0	4	2
DoMYB08	0	1	0	0	0	0	1	0	5	7	0	16	2	0	0	0	0	0	1	2	3
DoMYB09	3	3	0	1	3	0	1	0	1	2	0	6	4	2	0	0	0	3	2	6	4
DoMYB10	0	1	1	1	0	0	1	1	13	2	0	8	1	2	3	0	0	4	0	4	2
DoMYB100	0	4	0	0	0	0	0	0	1	0	0	13	0	2	1	0	0	0	0	1	3
DoMYB101	7	1	0	3	1	0	3	1	1	3	0	13	1	4	0	0	0	3	1	2	3
DoMYB102	0	1	0	0	0	0	0	0	2	7	0	24	0	6	4	0	0	3	0	1	2
DoMYB103	3	2	0	2	0	0	0	0	8	2	0	7	2	2	0	0	0	1	0	1	3
DoMYB104	0	0	0	0	0	0	1	0	3	3	0	11	1	2	0	0	0	0	0	1	1
DoMYB105	0	2	0	0	0	0	0	0	3	5	0	11	1	4	0	0	0	1	0	1	2
DoMYB106	5	1	0	1	0	0	0	0	4	1	0	7	2	2	2	0	0	0	1	1	2
DoMYB107	4	1	0	1	1	0	3	0	1	1	0	12	2	0	2	0	0	2	0	0	3
DoMYB108	0	2	0	0	0	0	0	0	1	1	0	14	0	2	1	0	0	3	0	2	3
DoMYB109	5	0	0	1	0	0	0	0	5	1	0	4	7	2	0	0	0	0	0	2	1
DoMYB11	0	5	0	0	0	0	0	0	7	3	0	4	0	0	0	0	0	1	2	2	2
DoMYB110	2	7	0	2	0	0	0	1	5	1	0	18	0	2	0	0	0	4	2	3	6
DoMYB111	4	2	0	0	0	0	1	1	0	3	0	8	0	2	2	0	2	0	0	0	3
DoMYB112	2	0	0	1	1	0	1	0	0	0	0	6	1	0	0	0	0	0	0	1	0
DoMYB113	0	0	0	0	0	0	0	0	0	0	0	1	0	0	0	0	0	0	0	0	0
DoMYB114	4	4	0	0	0	0	1	0	0	3	0	12	4	2	1	0	0	4	0	0	1
DoMYB115	0	1	0	0	0	0	1	0	0	1	0	4	0	2	0	0	0	1	0	2	2
DoMYB116	4	2	0	1	1	0	1	0	3	2	0	8	2	4	3	0	0	2	1	3	3
DoMYB117	0	0	0	0	0	0	0	1	2	1	0	5	2	4	0	0	0	1	0	0	1
DoMYB12	3	2	0	0	2	0	2	2	7	1	0	10	3	8	1	0	0	#	0	3	2
DoMYB13	4	2	0	0	0	0	0	0	0	2	0	5	1	0	2	0	0	1	2	2	2
DoMYB14	0	1	0	0	0	0	1	0	1	4	0	5	0	2	0	0	0	5	0	2	4
DoMYB15	7	4	0	0	0	0	2	1	0	1	0	10	2	0	2	0	0	1	1	4	5
DoMYB16	5	4	0	0	2	0	0	1	5	2	0	9	3	0	1	0	0	6	0	6	3
DoMYB17	0	0	0	1	1	0	1	0	2	2	0	6	2	2	1	0	0	5	0	0	3
DoMYB18	0	6	0	0	0	0	1	0	0	7	0	7	0	2	0	0	0	3	0	2	0
DoMYB19	8	2	0	0	2	0	0	0	1	5	0	12	5	2	1	0	0	5	0	2	2
DoMYB20	1	0	0	0	0	0	2	0	0	0	0	2	1	2	1	0	0	0	0	1	1
DoMYB21	0	5	0	0	0	0	1	1	3	1	0	17	0	4	0	0	0	1	0	1	2
DoMYB22	8	0	0	0	1	0	1	0	2	1	0	13	3	0	1	0	0	3	1	2	3
DoMYB23	3	1	0	1	0	0	1	1	5	1	0	19	1	2	0	0	0	5	2	3	2

DoMYB24	1	2	0	0	0	0	2	0	3	3	0	8	1	2	1	0	0	6	0	3	1
DoMYB25	4	2	0	0	1	0	0	0	3	5	0	17	2	0	0	0	0	0	0	0	1
DoMYB26	0	0	0	1	0	0	0	0	1	1	0	10	1	0	1	0	0	2	0	0	1
DoMYB27	3	2	0	1	0	0	2	0	4	5	0	7	1	2	0	0	0	2	0	4	3
DoMYB28	1	1	0	0	2	0	0	0	3	2	2	10	4	0	0	0	0	2	0	1	3
DoMYB29	3	0	0	0	3	0	0	0	4	1	0	10	4	4	2	1	0	7	0	2	2
DoMYB30	7	1	0	0	1	0	0	0	7	3	0	22	12	6	0	0	0	3	0	3	4
DoMYB31	7	4	0	0	1	0	2	0	2	2	0	7	2	0	2	0	0	2	0	2	1
DoMYB32	0	1	0	0	0	0	0	1	7	2	0	9	0	6	1	0	0	1	2	3	2
DoMYB33	5	0	0	0	0	0	1	0	3	4	0	8	0	4	0	0	0	1	0	0	3
DoMYB34	7	0	0	1	0	0	0	0	2	3	0	10	2	14	2	0	0	2	0	4	3
DoMYB35	2	1	0	0	2	0	0	1	1	3	0	17	2	2	0	0	0	1	0	1	2
DoMYB36	5	3	0	0	1	0	1	0	0	1	0	12	2	2	2	0	0	2	0	8	1
DoMYB37	0	1	0	1	0	0	3	0	1	5	0	2	2	10	1	0	2	1	0	4	5
DoMYB38	4	4	0	0	1	0	0	0	0	3	0	3	3	2	0	0	0	3	0	4	1
DoMYB39	2	2	0	0	1	0	0	0	1	2	0	6	2	2	0	0	0	1	0	1	2
DoMYB3R1	4	1	0	0	0	0	0	1	0	1	0	13	1	8	1	0	0	1	0	4	6
DoMYB3R2	6	5	0	1	2	0	0	2	1	2	0	5	4	0	1	0	0	3	1	3	0
DoMYB3R3	0	1	0	1	0	0	0	0	4	0	0	5	2	2	0	0	0	3	1	0	2
DoMYB3R4	3	1	0	0	1	0	0	0	1	4	0	8	1	0	1	0	0	2	0	2	2
DoMYB40	3	4	0	0	2	0	2	0	1	1	2	8	9	4	2	1	0	2	0	2	12
DoMYB41	5	1	0	1	0	0	2	0	9	1	0	26	0	2	1	0	0	0	1	2	3
DoMYB42	4	2	0	0	1	0	0	0	3	0	0	11	1	10	0	0	0	0	0	0	1
DoMYB43	1	4	0	0	1	0	3	0	1	4	2	4	10	4	0	0	0	3	1	1	6
DoMYB44	6	3	0	0	1	0	0	0	1	3	0	17	1	8	1	0	0	5	0	0	0
DoMYB45	1	3	0	0	1	0	1	0	3	2	0	7	2	2	1	0	0	3	1	3	4
DoMYB46	5	2	0	0	2	0	0	0	6	1	2	13	4	4	1	0	0	3	0	4	2
DoMYB47	19	2	0	1	0	0	1	0	3	3	0	25	1	2	0	0	0	2	0	2	1
DoMYB48	1	1	0	0	1	0	0	1	5	1	0	8	2	6	1	0	0	3	0	1	4
DoMYB49	3	1	0	0	1	0	0	0	4	1	0	17	3	2	0	0	0	2	0	1	0
DoMYB4R1	4	4	0	0	0	0	1	1	2	1	0	12	0	0	0	0	0	1	0	0	0
DoMYB50	0	0	0	2	0	0	0	0	2	2	0	6	2	4	0	0	0	0	0	1	0
DoMYB51	13	1	0	1	0	1	2	0	1	3	0	16	1	6	0	0	0	3	0	7	2
DoMYB52	7	1	0	1	0	0	0	0	3	3	0	6	1	4	0	0	0	3	0	2	1
DoMYB53	1	1	0	2	1	0	1	0	3	1	0	8	4	6	4	0	0	1	1	2	2
DoMYB54	2	0	0	0	0	0	0	1	3	1	0	18	0	0	1	0	0	0	1	1	3
DoMYB55	2	4	0	1	3	0	2	1	3	3	0	7	3	4	0	0	0	0	0	2	0
DoMYB56	0	2	0	1	1	0	1	0	0	5	0	11	2	0	1	0	0	3	0	3	0
DoMYB57	2	1	0	0	1	0	0	0	3	3	0	15	1	2	1	0	2	2	0	1	2
DoMYB58	0	1	0	0	0	0	0	1	1	2	0	1	0	0	0	0	0	0	0	0	0
DoMYB59	12	1	0	1	0	0	1	0	3	2	0	13	1	4	2	0	0	0	0	5	2
DoMYB60	2	6	0	1	0	0	2	0	1	2	0	10	0	6	1	0	0	3	0	6	3
DoMYB61	4	2	0	0	2	0	1	1	0	3	0	10	2	2	0	0	0	1	1	1	3
DoMYB62	3	3	0	1	2	0	1	0	3	3	0	13	4	2	1	0	2	1	1	5	1

DoMYB63	3	2	0	1	0	0	1	0	0	2	2	2	4	4	0	0	0	4	0	5	1
DoMYB64	10	1	0	1	2	0	2	0	1	3	0	20	3	0	0	0	2	0	1	2	2
DoMYB65	0	3	0	1	0	0	2	1	2	2	0	11	0	4	0	0	0	2	0	1	0
DoMYB66	0	5	0	0	0	0	1	0	3	0	0	10	0	4	1	0	0	1	0	4	2
DoMYB67	2	1	0	1	0	0	0	0	3	0	0	8	1	6	0	0	0	2	0	5	3
DoMYB68	5	2	0	4	2	0	1	2	2	0	2	6	4	14	4	0	0	4	0	2	5
DoMYB69	4	2	0	1	0	0	2	0	3	1	2	3	3	4	2	0	0	2	0	3	2
DoMYB70	4	2	0	1	1	0	0	0	0	2	0	16	3	0	0	0	0	0	2	2	2
DoMYB71	5	3	0	1	0	0	1	1	8	3	0	31	1	2	1	0	0	5	1	2	3
DoMYB72	1	0	0	0	1	0	0	0	2	2	0	7	3	2	1	0	0	2	0	2	2
DoMYB73	4	1	0	0	0	0	1	0	4	2	0	19	1	0	2	0	0	1	0	1	4
DoMYB74	1	3	0	1	1	0	0	0	2	1	0	12	1	4	1	0	0	5	0	2	4
DoMYB75	2	3	0	0	3	0	1	0	3	2	0	6	5	6	1	0	0	0	0	1	0
DoMYB76	0	2	0	0	0	0	2	1	2	1	0	10	0	0	2	0	0	0	0	1	2
DoMYB77	4	0	0	0	0	0	0	0	2	3	0	4	0	4	2	0	0	1	0	2	3
DoMYB78	6	0	0	2	0	0	2	1	2	0	0	11	1	18	3	0	0	2	0	2	2
DoMYB79	4	0	0	0	3	0	0	1	3	1	0	8	9	6	0	0	0	4	0	1	1
DoMYB80	10	0	0	0	3	0	1	0	4	3	0	16	5	0	1	0	0	1	0	1	2
DoMYB81	7	1	0	0	0	0	1	0	1	10	0	12	0	6	1	1	0	3	0	2	1
DoMYB82	10	4	0	1	0	0	0	0	4	1	0	27	0	4	0	0	2	1	0	1	2
DoMYB83	0	0	0	0	0	0	0	1	2	2	0	14	0	2	2	0	0	2	0	0	3
DoMYB84	5	3	0	0	2	0	1	2	2	0	0	4	8	6	2	0	0	2	0	3	1
DoMYB85	2	0	0	0	0	0	0	1	4	1	0	6	5	0	1	0	0	4	0	2	10
DoMYB86	2	0	0	1	0	0	0	0	0	1	0	5	1	0	0	0	0	0	0	0	2
DoMYB87	2	2	0	2	0	0	0	1	0	3	0	21	0	4	0	1	0	1	0	4	0
DoMYB88	8	2	0	2	2	0	1	0	2	5	0	16	4	4	0	0	0	1	0	1	6
DoMYB89	2	2	0	0	2	0	1	0	3	0	0	7	2	6	1	0	0	3	0	1	1
DoMYB90	4	2	0	0	1	0	2	0	3	1	0	18	5	0	2	0	6	0	0	0	2
DoMYB91	8	1	0	0	0	0	0	0	3	3	0	12	0	0	1	0	0	2	0	3	2
DoMYB92	10	2	0	0	2	0	0	0	2	2	0	24	4	4	3	0	0	1	0	3	1
DoMYB93	8	2	0	0	2	0	0	0	1	2	0	21	3	2	1	0	0	5	0	0	3
DoMYB94	1	0	0	0	1	0	0	0	7	4	0	9	1	4	0	0	0	0	0	0	1
DoMYB95	2	2	0	0	0	0	1	0	6	2	0	8	1	0	0	0	0	1	0	6	2
DoMYB96	14	1	0	2	0	1	1	0	3	1	0	10	3	2	2	0	0	2	0	6	2
DoMYB97	6	3	0	1	0	0	1	0	0	1	0	10	0	2	2	0	0	1	0	1	4
DoMYB98	7	1	0	0	0	0	0	0	6	0	2	15	2	4	0	0	0	5	0	1	3
DoMYB99	6	1	0	0	1	0	0	1	3	3	0	10	1	0	2	0	0	1	0	5	0
DoMYBR01	8	3	0	3	1	0	2	0	0	3	0	4	2	2	3	0	0	2	1	5	3
DoMYBR02	9	0	0	1	0	0	1	0	4	1	4	7	3	4	1	0	0	6	0	1	2
DoMYBR03	5	0	1	1	0	0	0	0	4	1	0	15	10	6	1	0	0	0	0	1	5
DoMYBR04	2	1	0	0	0	0	0	0	3	0	0	5	0	4	0	0	0	2	0	2	2
DoMYBR05	3	2	0	0	3	0	3	0	1	4	0	9	4	0	3	0	0	2	0	2	0
DoMYBR06	11	1	0	1	0	0	1	0	4	1	0	16	3	8	0	0	0	0	0	0	2
DoMYBR07	0	5	0	0	0	0	1	1	1	4	0	8	2	14	0	0	0	4	0	1	2

DoMYBR08	2	4	0	1	0	0	2	0	2	0	2	11	1	0	2	0	0	0	0	3	1
DoMYBR09	0	1	0	1	0	0	1	0	0	4	0	8	3	2	2	0	0	2	0	5	1
DoMYBR10	2	1	1	2	0	0	1	0	1	4	0	8	0	0	0	0	0	0	0	1	0
DoMYBR11	1	1	0	2	0	0	1	0	2	2	0	5	7	12	3	0	0	5	0	3	1
DoMYBR12	2	1	0	0	1	0	1	0	1	1	0	8	1	0	0	0	0	1	0	2	2
DoMYBR13	12	2	0	1	0	0	0	0	4	3	0	12	3	6	2	0	0	4	0	1	2
DoMYBR14	4	3	0	0	2	0	1	0	5	1	0	16	7	4	0	0	0	3	0	2	0
DoMYBR15	5	2	0	1	1	0	3	1	6	1	0	19	2	8	1	0	0	2	1	2	2
DoMYBR16	0	0	0	0	0	0	0	0	3	1	0	16	0	2	1	0	0	3	0	1	1
DoMYBR17	8	1	0	2	6	0	0	0	3	0	0	10	19	32	1	0	0	3	1	1	1
DoMYBR18	0	1	0	0	0	0	1	0	4	1	0	36	1	2	1	0	0	5	0	1	1
DoMYBR19	6	4	0	2	3	0	0	0	1	2	0	8	9	8	1	0	0	3	0	5	3
DoMYBR20	5	3	0	0	0	0	1	0	2	6	0	17	1	8	0	0	0	1	0	2	1
DoMYBR21	4	1	0	1	2	0	1	0	3	3	0	14	10	2	0	0	0	3	0	1	4
DoMYBR22	8	3	0	1	1	0	0	1	3	0	0	6	4	6	1	0	0	1	0	2	5
DoMYBR23	5	0	0	1	5	0	3	0	0	1	0	7	9	10	3	0	0	5	0	2	5
DoMYBR24	0	3	0	1	0	0	0	0	4	3	0	12	1	0	1	0	0	5	0	3	1
DoMYBR25	3	2	0	0	0	0	1	0	4	5	0	23	0	2	1	0	0	1	1	1	3
DoMYBR26	0	1	0	0	1	0	0	0	8	2	0	10	2	4	0	0	2	3	0	3	0
DoMYBR27	6	1	0	2	0	0	1	0	1	2	0	11	2	8	0	0	0	2	1	0	1
DoMYBR28	3	2	0	0	1	0	0	2	7	2	0	4	2	6	0	0	0	3	1	3	2
DoMYBR29	4	4	0	2	1	0	1	0	2	2	0	7	5	8	0	0	0	2	0	4	2
DoMYBR30	0	1	0	0	0	0	1	0	4	6	0	5	0	0	2	0	0	2	0	2	3
DoMYBR31	0	2	0	0	0	0	1	0	3	2	0	10	0	0	1	0	0	3	0	1	4
DoMYBR32	4	2	1	2	2	0	1	0	5	2	0	13	2	2	0	0	0	1	1	1	3
DoMYBR33	1	3	0	0	2	0	1	0	2	1	0	7	7	0	1	0	2	2	0	2	2
DoMYBR34	6	2	0	2	3	0	0	0	5	1	0	9	5	2	1	0	0	3	1	2	4
DoMYBR35	8	4	0	1	1	0	1	0	6	1	0	14	1	2	1	0	0	3	0	2	2
DoMYBR36	3	1	0	0	1	0	0	0	0	2	0	6	2	2	3	0	0	0	0	1	3
DoMYBR37	5	0	0	0	0	0	1	0	3	4	0	11	0	0	0	0	0	4	0	3	5
DoMYBR38	3	1	0	0	0	0	0	0	18	1	0	17	0	0	0	0	0	4	0	1	1
DoMYBR39	6	4	0	1	1	0	1	0	8	2	0	11	3	2	1	0	0	0	0	0	3
DoMYBR40	9	1	0	1	4	0	0	0	2	2	0	11	9	2	1	0	0	3	0	1	5
DoMYBR41	0	0	0	0	0	0	0	0	0	1	0	1	0	0	0	0	0	0	0	0	0
DoMYBR42	0	5	0	1	1	0	1	0	1	3	0	11	4	4	1	0	0	4	0	1	2
PeMYB01	2	2	0	0	0	0	0	0	2	4	0	9	3	2	1	1	0	5	0	3	4
PeMYB02	3	1	0	0	0	0	0	1	0	0	0	16	1	0	1	0	0	6	1	4	2
PeMYB03	2	2	0	2	0	0	1	0	4	1	0	9	1	4	0	0	0	3	1	2	5
PeMYB04	11	0	0	1	0	0	0	0	1	2	0	21	1	4	1	0	0	0	0	0	2
PeMYB05	3	1	0	1	1	0	0	0	7	1	2	17	1	6	0	1	2	1	0	0	3
PeMYB06	3	2	0	0	0	0	1	0	1	1	0	6	0	4	0	0	2	1	0	1	1
PeMYB07	8	1	0	1	0	0	1	0	0	2	0	9	1	2	1	0	0	0	0	1	2
PeMYB08	5	1	0	2	2	0	4	0	1	3	0	10	2	6	2	0	0	3	0	3	5
PeMYB09	10	0	0	1	2	0	1	0	0	6	0	14	9	6	0	0	6	2	0	3	4

PeMYB10	7	2	0	0	1	0	0	0	4	4	0	11	2	2	1	0	0	4	1	4	2
PeMYB100	0	2	0	0	0	0	0	0	1	0	0	4	0	0	0	0	0	1	0	1	0
PeMYB101	0	0	0	0	0	0	0	0	0	0	0	1	0	0	0	0	0	2	0	2	0
PeMYB102	0	0	0	1	0	0	2	0	5	1	0	14	0	2	1	0	0	3	0	1	3
PeMYB103	1	3	0	0	1	0	0	0	3	3	0	11	2	0	0	0	0	3	0	3	3
PeMYB104	6	4	0	1	0	0	0	2	5	4	0	5	1	0	0	0	0	1	1	2	6
PeMYB105	4	1	0	0	0	0	1	0	7	0	0	16	0	4	0	0	0	5	2	6	2
PeMYB106	9	5	0	1	0	0	0	0	3	3	0	11	1	4	1	0	0	2	1	4	0
PeMYB107	4	6	0	0	2	0	2	0	0	2	0	16	5	0	1	0	0	2	1	1	1
PeMYB108	0	0	0	0	1	0	1	0	7	6	0	3	1	2	1	0	0	3	1	0	0
PeMYB109	2	0	1	0	0	0	0	0	0	1	0	3	1	2	0	0	0	0	0	0	0
PeMYB11	2	3	0	1	0	0	0	0	2	1	0	12	2	0	1	0	0	0	0	2	0
PeMYB110	0	2	0	1	1	0	1	0	3	4	0	13	1	2	0	0	0	0	0	1	2
PeMYB111	1	0	0	0	0	0	2	0	3	1	0	7	5	0	1	0	0	1	0	2	3
PeMYB112	10	4	0	0	0	0	0	0	5	0	0	25	0	4	0	0	0	2	0	5	2
PeMYB113	18	2	0	1	0	0	2	0	1	3	0	11	3	2	1	0	0	3	0	4	1
PeMYB114	12	1	0	2	2	0	1	3	4	3	0	18	3	4	3	0	0	1	0	1	5
PeMYB115	9	4	0	0	0	0	0	0	1	0	0	13	0	4	0	0	0	0	0	3	0
PeMYB12	4	4	0	0	0	0	2	0	4	2	0	10	1	0	0	0	0	1	0	2	4
PeMYB13	4	2	0	0	0	0	1	0	2	1	0	10	0	0	1	0	4	1	1	2	5
PeMYB14	1	2	0	0	0	0	2	0	2	1	0	5	0	8	0	0	0	4	1	3	0
PeMYB15	13	2	0	0	0	0	1	0	1	3	0	21	0	6	1	1	0	4	1	4	1
PeMYB16	3	4	0	0	0	0	1	0	3	3	0	12	1	0	0	0	0	2	1	1	2
PeMYB17	7	2	0	1	1	0	0	0	6	1	2	18	2	4	1	0	0	6	0	2	2
PeMYB18	3	0	0	1	0	0	0	2	1	4	0	16	0	2	0	0	0	3	0	1	3
PeMYB19	0	0	0	0	0	0	0	0	1	1	0	8	0	0	1	0	2	0	0	0	5
PeMYB20	14	3	0	3	5	0	1	0	1	1	0	15	7	14	0	0	0	5	0	3	4
PeMYB21	18	0	0	0	0	1	1	0	3	5	0	29	1	2	2	0	0	1	1	3	5
PeMYB22	3	4	0	0	0	0	1	0	3	2	0	18	1	4	0	0	0	1	0	2	4
PeMYB23	2	2	0	1	0	0	1	0	4	2	0	15	0	0	4	0	0	4	0	6	1
PeMYB24	1	1	0	0	0	0	0	1	2	3	0	18	0	2	1	0	0	1	0	2	2
PeMYB25	15	3	0	0	0	0	4	0	1	6	0	16	0	8	1	0	0	3	0	6	5
PeMYB26	7	1	0	0	2	0	0	0	0	0	2	7	4	6	1	0	2	2	0	3	0
PeMYB27	4	0	0	0	0	0	1	0	3	1	0	14	0	8	4	0	0	0	0	2	1
PeMYB28	4	2	0	1	1	0	0	0	0	4	0	9	3	4	0	0	0	0	0	2	1
PeMYB29	6	0	0	1	1	0	4	0	2	0	0	10	4	8	2	0	0	7	0	3	4
PeMYB30	14	3	0	1	1	0	0	1	5	3	0	10	2	2	0	0	0	2	0	3	4
PeMYB31	3	3	0	3	1	0	0	0	6	1	0	29	6	6	3	1	0	2	0	1	2
PeMYB32	15	4	0	1	0	0	0	0	4	6	0	12	0	4	2	0	2	3	0	3	2
PeMYB33	6	1	0	1	0	0	1	1	1	4	0	11	1	2	0	0	2	2	1	0	1
PeMYB34	3	3	0	1	0	0	1	0	8	4	0	7	1	2	2	0	0	1	1	3	8
PeMYB35	5	0	0	0	1	0	1	0	2	1	0	7	1	0	2	0	0	2	0	5	4
PeMYB36	11	4	0	0	0	0	2	2	3	2	0	18	0	0	1	0	2	2	0	4	2
PeMYB37	1	1	0	1	1	0	1	0	3	4	0	6	3	4	3	0	0	2	0	3	0

PeMYB38	14	1	0	0	4	0	2	0	2	1	0	11	6	0	3	0	2	7	0	4	4
PeMYB39	2	1	0	1	0	0	0	2	2	2	0	7	0	4	0	0	0	4	0	3	3
PeMYB3R1	1	1	0	1	1	0	1	0	2	2	0	11	5	2	0	0	0	3	0	5	3
PeMYB3R2	0	3	0	1	0	0	1	0	3	2	0	10	0	2	0	0	2	2	0	2	2
PeMYB3R3	9	2	0	1	0	0	0	1	3	3	0	15	2	6	1	0	2	1	0	8	1
PeMYB40	3	2	0	0	0	0	0	0	1	0	0	12	0	0	1	0	0	2	0	2	3
PeMYB41	13	3	0	0	1	0	1	0	4	4	0	13	1	6	0	0	0	2	1	4	3
PeMYB42	2	3	0	1	0	0	0	2	5	2	0	6	0	10	0	0	0	0	0	1	6
PeMYB43	14	5	0	0	2	0	2	1	2	0	0	6	6	14	1	0	0	5	0	4	1
PeMYB44	3	2	0	0	1	0	0	2	4	4	2	14	6	2	0	0	0	2	0	4	5
PeMYB45	11	1	0	0	2	0	0	0	4	2	0	10	4	4	1	0	0	2	0	1	2
PeMYB46	4	0	0	1	0	0	1	0	2	5	0	7	0	2	0	0	0	0	0	5	1
PeMYB47	4	1	0	1	0	0	0	0	7	1	0	18	5	2	1	0	0	0	0	3	0
PeMYB48	0	1	0	1	0	0	1	0	3	3	0	7	1	2	0	0	2	4	0	5	2
PeMYB49	1	1	0	2	0	0	1	0	2	4	0	7	3	6	2	0	0	0	0	3	1
PeMYB50	3	3	1	0	0	0	1	1	2	2	0	1	0	4	1	0	0	3	0	1	0
PeMYB51	5	2	0	1	0	0	3	0	1	3	0	7	0	0	0	0	0	3	0	3	3
PeMYB52	0	0	0	1	0	0	1	0	1	3	0	4	0	2	2	0	0	3	0	2	0
PeMYB53	5	0	0	1	4	0	3	0	2	2	0	13	4	0	1	0	0	1	0	3	1
PeMYB54	0	2	0	0	2	0	0	0	4	3	0	3	5	0	2	0	0	3	0	4	0
PeMYB55	6	2	0	1	0	0	0	0	2	1	0	16	1	0	2	0	0	6	0	2	1
PeMYB56	6	3	0	0	1	0	2	1	3	0	0	11	2	6	0	0	0	5	0	1	0
PeMYB57	3	3	0	2	1	0	2	0	3	5	0	8	2	0	0	0	0	1	0	2	0
PeMYB58	0	1	0	1	1	0	0	0	2	3	0	17	1	2	0	0	0	1	0	1	3
PeMYB59	6	3	0	1	0	0	0	0	3	4	0	14	6	2	0	0	0	1	0	2	2
PeMYB60	0	0	0	0	0	0	0	0	2	3	0	4	2	2	0	1	0	1	1	1	0
PeMYB61	4	0	0	1	0	0	0	0	2	3	0	10	2	2	2	0	0	2	0	2	3
PeMYB62	3	1	0	1	2	0	2	0	0	0	0	5	4	6	3	0	0	4	0	2	3
PeMYB63	2	3	0	0	1	0	1	0	4	5	0	12	3	0	1	0	0	3	0	2	3
PeMYB64	2	1	0	0	0	0	2	0	0	1	0	4	0	0	1	2	0	2	0	2	0
PeMYB65	1	7	0	0	0	0	2	0	6	2	0	8	2	0	1	0	0	5	1	6	2
PeMYB66	4	1	0	0	0	0	1	0	2	2	0	5	6	0	2	0	0	4	2	2	1
PeMYB67	6	0	0	0	0	0	2	1	4	4	0	12	0	2	0	0	0	0	1	1	1
PeMYB68	5	3	0	2	0	0	1	0	4	2	0	16	2	6	5	0	0	2	0	1	2
PeMYB69	0	6	0	1	0	0	0	0	10	2	0	8	1	2	0	0	0	3	0	3	3
PeMYB70	0	0	0	0	0	0	0	0	0	4	0	12	1	0	0	0	0	1	0	1	0
PeMYB71	5	3	0	1	0	0	1	0	2	3	0	9	1	2	0	0	0	1	1	3	1
PeMYB72	0	1	0	1	0	0	0	0	2	5	0	13	0	2	0	1	0	2	0	4	4
PeMYB73	7	1	0	0	1	0	0	0	4	2	0	17	2	2	1	0	0	1	1	2	0
PeMYB74	6	3	0	0	0	0	2	0	0	0	0	10	0	4	0	0	0	3	0	5	2
PeMYB75	5	3	0	1	4	0	0	0	4	3	0	11	7	4	1	1	2	1	1	1	2
PeMYB76	0	3	0	0	0	0	0	0	3	3	0	12	0	0	0	0	0	2	0	3	5
PeMYB77	5	2	0	0	0	0	1	0	1	1	0	7	0	2	0	0	0	1	1	2	0
PeMYB78	2	0	0	1	0	0	1	1	1	2	0	25	0	0	2	1	0	1	0	4	1

PeMYB79	5	4	0	2	2	0	1	0	1	3	0	18	2	4	0	0	0	0	0	2	0
PeMYB80	5	3	0	0	3	0	2	0	4	3	0	12	4	4	1	0	0	1	0	1	4
PeMYB81	4	0	0	0	3	0	1	0	3	0	0	7	6	0	1	0	0	2	0	2	2
PeMYB82	2	1	0	0	0	0	3	0	0	1	0	4	0	0	1	2	0	2	0	3	0
PeMYB83	2	2	1	0	0	0	2	1	5	2	0	3	0	4	2	0	0	5	0	1	1
PeMYB84	0	2	0	0	0	0	0	0	1	1	0	6	3	2	1	1	0	4	0	3	3
PeMYB85	5	0	0	0	0	0	1	0	0	0	0	5	0	4	1	0	0	0	0	1	1
PeMYB86	0	2	0	4	0	0	2	2	2	2	0	7	0	0	0	0	0	2	0	2	0
PeMYB87	0	2	0	4	0	0	2	2	2	2	0	6	0	0	0	0	0	2	0	2	0
PeMYB88	5	1	0	0	1	0	2	1	3	6	0	15	2	2	3	0	0	4	0	2	2
PeMYB89	9	3	0	1	0	0	1	0	2	4	0	10	1	2	2	0	0	9	0	5	0
PeMYB90	4	0	0	0	0	0	1	0	1	4	0	14	1	2	0	0	0	2	0	1	3
PeMYB91	9	2	0	0	0	0	0	0	2	0	0	8	1	2	0	0	0	3	0	0	4
PeMYB92	0	1	0	1	0	0	0	0	2	3	0	12	0	0	0	1	0	2	0	4	4
PeMYB93	0	2	0	0	0	0	1	0	2	3	0	14	1	0	1	0	0	0	0	1	5
PeMYB94	0	0	0	0	0	0	0	0	0	0	0	0	0	0	0	0	0	0	0	1	1
PeMYB95	0	3	0	0	0	0	1	0	2	2	0	10	1	0	0	0	0	0	0	1	4
PeMYB96	2	0	0	1	1	0	0	0	0	0	0	1	2	0	1	0	0	1	0	0	0
PeMYB97	1	5	0	2	2	0	1	0	3	1	0	11	3	4	0	0	0	3	0	5	3
PeMYB98	0	1	0	1	0	0	2	0	0	4	0	14	1	2	0	0	0	4	0	2	1
PeMYB99	3	1	0	1	0	0	0	0	0	0	0	2	0	0	0	0	0	0	0	0	0
PeCDC	0	6	0	2	3	0	1	0	1	1	0	7	7	6	3	0	0	7	0	1	4
PeMYBR01	11	3	0	1	0	0	1	0	0	2	0	15	4	2	1	0	0	5	0	0	2
PeMYBR02	9	2	0	1	1	0	1	0	1	2	0	9	4	0	0	0	0	3	0	2	1
PeMYBR03	1	1	0	3	0	0	1	0	5	3	0	15	1	2	3	0	0	2	0	2	2
PeMYBR04	0	1	0	1	0	0	1	0	2	2	0	7	1	0	2	0	0	1	1	5	2
PeMYBR05	0	0	0	2	0	0	0	0	0	1	0	3	2	2	0	0	0	3	0	1	1
PeMYBR06	22	3	0	0	0	0	0	0	5	0	2	21	4	6	3	0	0	3	0	0	3
PeMYBR07	0	0	0	0	0	0	1	0	4	2	0	5	2	4	1	0	0	1	1	1	2
PeMYBR08	7	4	0	0	1	0	3	0	0	0	0	11	8	6	2	0	0	2	0	4	4
PeMYBR09	11	0	0	0	1	0	0	0	5	0	0	10	4	6	1	0	0	5	0	2	2
PeMYBR10	4	2	0	3	0	0	1	0	0	2	0	10	3	4	1	0	0	2	0	2	4
PeMYBR11	9	4	0	2	2	0	0	0	1	3	0	23	4	2	0	0	0	2	0	5	0
PeMYBR12	4	1	0	1	1	0	0	0	2	1	0	10	3	2	3	0	0	3	0	3	2
PeMYBR13	7	1	0	2	1	0	5	1	2	1	0	9	2	6	2	0	0	1	0	2	1
PeMYBR14	3	2	2	0	0	0	0	0	4	2	0	20	1	2	0	1	0	2	0	2	2
PeMYBR15	2	0	0	1	0	0	1	0	2	2	0	23	2	2	0	0	0	2	1	2	5
PeMYBR16	5	3	0	0	0	0	3	0	3	1	0	10	3	2	0	0	0	2	1	3	3
PeMYBR17	3	4	0	0	1	0	0	0	5	2	0	11	1	0	1	1	0	3	0	2	2
PeMYBR18	13	2	0	1	1	0	2	0	2	4	0	11	3	2	2	0	0	6	0	2	3
PeMYBR19	1	1	0	1	0	0	1	0	2	0	0	12	2	0	1	0	0	1	0	4	1
PeMYBR20	0	3	0	1	0	0	0	0	1	1	0	14	2	4	2	0	0	2	0	3	3
PeMYBR21	0	0	0	0	0	0	0	0	2	3	0	3	0	0	0	0	0	2	0	2	0
PeMYBR22	3	3	0	0	1	0	1	1	4	5	0	6	1	2	1	0	0	1	0	2	2

PeMYBR23	6	2	0	0	0	0	0	0	6	0	0	10	0	4	1	0	0	6	0	3	5
PeMYBR24	1	1	0	1	0	0	4	0	2	0	2	3	2	2	0	0	0	1	0	4	8
PeMYBR25	5	3	0	0	2	0	0	0	1	3	0	12	8	4	2	0	0	2	0	3	2
PeMYBR26	5	3	0	1	2	0	1	1	0	3	0	15	3	2	0	1	0	2	2	2	5
PeMYBR27	2	1	0	0	1	0	0	1	0	2	0	14	3	2	3	0	0	4	0	3	3
PeMYBR28	5	2	0	0	0	0	1	0	5	5	0	18	1	6	1	0	0	0	0	2	1
PeMYBR29	1	5	0	1	0	0	1	0	5	2	0	8	2	8	0	0	0	0	1	3	0
PeMYBR30	6	4	1	0	1	0	0	0	3	4	0	8	5	2	1	0	0	4	0	1	0
PeMYBR31	1	2	0	1	1	0	0	0	1	4	0	15	2	2	0	0	0	0	1	0	0
PeMYBR32	4	2	0	1	0	0	3	0	1	3	0	5	1	8	2	0	0	0	0	3	7
PeMYBR33	1	1	0	0	0	0	0	0	2	2	0	8	0	0	1	0	0	3	1	2	4
PeMYBR34	0	2	0	0	0	0	0	0	3	6	0	6	1	2	2	0	0	2	1	0	2
PeMYBR35	1	1	0	1	1	0	3	0	1	3	0	5	2	2	4	0	0	1	0	1	3
PeMYBR36	3	1	0	1	0	0	0	0	2	4	0	6	0	0	1	0	2	1	1	1	4
PeMYBR37	5	4	0	0	2	0	2	1	2	5	2	7	5	6	1	0	0	3	0	3	3
PeMYBR38	0	3	0	0	0	0	1	0	0	6	0	6	0	2	1	0	0	2	0	2	4
PeMYBR39	1	0	0	0	1	0	0	0	0	0	0	7	1	0	0	0	0	1	0	0	1
PeMYBR40	2	5	0	2	0	0	1	0	1	4	0	5	0	6	0	0	0	3	0	4	5

A, ABA response element; B, anaerobic induction element; C, antioxidant response element; D, auxin response element; E, dehydration response element; F, desiccation response element; G, drought response element; H, endosperm-specific element; I, ethylene response element; J, GA response element; K, heat response element; L, light response element; M, low temperature response element; N, MeJA response element; O, meristem-specific element; P, SA response element; Q, seed-specific element; R, stress response element; S, sucrose response element; T, sulfur response element; U, wound

Supplementary Table 4 the FPKM of MYB genes in *D. officinale* under control and cold stress treatment.

Gene name	control (FPKM mean)	cold (FPKM mean)	fold change	regulation
DoMYB01	11.91513	0.118333	0.009931325	down
DoMYB02	0.666866	0.361887667		
DoMYB03	0.020403	0		
DoMYB04	1.010758	0.571886333		
DoMYB05	15.44754	11.36099533	0.735456637	
DoMYB06	0.36341	0		
DoMYB07	1.94824	32.87629933	16.87487134	up
DoMYB08	0	0		
DoMYB09	1.385159	1.678637		
DoMYB10	1.060093	0.235188		
DoMYB100	0.146837	0.046790667		
DoMYB101	0.103826	0.148914		
DoMYB102	0	0		
DoMYB103	0.856707	1.095761		
DoMYB104	0.026312	0		
DoMYB105	0.072693	0.238667667		
DoMYB106	0.063829	0.249320667		
DoMYB107	0.374449	0.195160667		
DoMYB108	0.026259	0		
DoMYB109	0.013029	0		
DoMYB11	0.087264	0.449784		
DoMYB110	1.294131	0.868285		
DoMYB111	1.713429	0.250477667		
DoMYB112	0.244597	0.485908		
DoMYB113	0	0		
DoMYB114	0.422021	0.146169		
DoMYB115	0.019503	0.022014333		
DoMYB116	0	0		
DoMYB117	0.089313	0		
DoMYB12	0	0		
DoMYB13	0	0		
DoMYB14	0.344993	0.493911333		
DoMYB15	0	0		
DoMYB16	0.300631	0.182201		
DoMYB17	0	0.140103		
DoMYB18	16.06584	16.83182933	1.047678116	
DoMYB19	0.506196	4.358088667		
DoMYB20	0.604578	0.505372		
DoMYB21	0.794091	0.486185		
DoMYB22	13.48476	5.086476333	0.377201974	down
DoMYB23	0	0		

DoMYB24	70.32641	100.858144	1.434143171	
DoMYB25	2.721632	1.215991667		
DoMYB26	0	0		
DoMYB27	0.360595	0.879125667		
DoMYB28	3.206202	0.041530333		
DoMYB29	0.313713	0.245775667		
DoMYB30	0.446483	0.804536333		
DoMYB31	0.007613	0.059619667		
DoMYB32	4.946932	5.346619667	1.080794987	
DoMYB33	12.19826	23.03153067	1.888099411	up
DoMYB34	2.562184	1.755991		
DoMYB35	0.665683	0.528305		
DoMYB36	0.011951	0		
DoMYB37	13.16544	5.900471	0.448178645	down
DoMYB38	0.015911	0.031831667		
DoMYB39	7.531142	5.316149667	0.705888948	
DoMYB40	2.387278	0.36708		
DoMYB41	6.206526	0.681182667	0.109752643	down
DoMYB42	0.700228	0.003421667		
DoMYB43	0.030281	0.02818		
DoMYB44	0	0		
DoMYB45	0.012897	0.103645		
DoMYB46	0.36427	0.095712333		
DoMYB47	0.223666	0.206817667		
DoMYB48	45.32693	44.470937	0.981115221	
DoMYB49	0	0.012395		
DoMYB50	1.83219	1.343438333		
DoMYB51	11.39035	2.299554667	0.201886205	down
DoMYB52	0	0.053177667		
DoMYB53	0	0.049312333		
DoMYB54	1.01794	0.459203		
DoMYB55	0.274025	0.279912667		
DoMYB56	0.057666	0.113238667		
DoMYB57	4.228115	0.470666		
DoMYB58	0	0		
DoMYB59	0.086154	0.202814333		
DoMYB60	17.14127	18.896438	1.102394602	
DoMYB61	1.275361	1.319464		
DoMYB62	0	0		
DoMYB63	0.059587	0.011201667		
DoMYB64	0.781739	0.453444333		
DoMYB65	20.17128	19.617451	0.972543814	
DoMYB66	1.152953	0		
DoMYB67	0	0.224123		

DoMYB68	0.786428	0.338531333		
DoMYB69	12.0084	19.36787167	1.612860303	up
DoMYB70	0	0		
DoMYB71	0	0		
DoMYB72	12.84657	15.65906633	1.218929369	
DoMYB73	4.45579	1.477052		
DoMYB74	0	0		
DoMYB75	1.505585	3.705206		
DoMYB76	0.308601	0.408458333		
DoMYB77	1.25036	0.576904		
DoMYB78	1.657842	1.648238		
DoMYB79	0.067045	0.058691		
DoMYB80	41.75501	34.871119	0.835136087	
DoMYB81	2.092994	0.50604		
DoMYB82	1.149594	0.321553667		
DoMYB83	0	0		
DoMYB84	0.232648	0.641845333		
DoMYB85	27.147	29.85726867	1.099836603	
DoMYB86	0	0		
DoMYB87	0	0		
DoMYB88	0	0		
DoMYB89	0	0		
DoMYB90	0.32497	0.581812667		
DoMYB91	14.11995	12.892374	0.91306099	
DoMYB92	2.334031	0.300357667		
DoMYB93	1.848708	2.030009667		
DoMYB94	4.513987	2.146758		
DoMYB95	0.16939	0.06831		
DoMYB96	9.386499	0.805106667	0.085772836	down
DoMYB97	0	0.024092333		
DoMYB98	0.109703	0.08022		
DoMYB99	0.016561	0.032493667		
DoMYBR01	10.97013	9.279648667	0.84590156	
DoMYBR02	56.34755	200.2195667	3.553296757	up
DoMYBR03	46.82462	68.38963333	1.460548707	
DoMYBR04	47.14741	120.0275667	2.545793431	up
DoMYBR05	71.84823	92.68196333	1.289968637	
DoMYBR07	12.25701	16.57957667	1.352660703	
DoMYBR08	39.23765	36.49246667	0.93003701	
DoMYBR09	29.74368	12.46062833	0.4189336	down
DoMYBR11	10.38622	7.060438	0.679789273	
DoMYBR12	12.08167	5.795462	0.479690606	down
DoMYBR17	29.0051	25.09219	0.865095694	
DoMYBR20	43.55513	79.58014667	1.827112916	up

DoMYBR23	95.1346	94.27959667	0.991012698	
DoMYBR24	32.15451	37.02902	1.151596463	
DoMYBR26	14.44546	20.18362333	1.397229856	
DoMYBR27	6.694202	145.8850667	21.79275049	up
DoMYBR29	2.780317	3.757446333		
DoMYBR32	1.261351	1.935758		
DoMYBR33	3.150581	4.083642333		
DoMYBR34	15.38062	128.8161	8.37521973	up
DoMYBR36	14.09781	30.76883	2.182525152	up
DoMYBR38	0.639016	0.225987333		
DoMYBR41	0	0		
DoMYBR42	3.547907	6.694379667	1.886853197	up
DoMYBR15	7.516451	9.644825	1.28316215	
DoMYBR21	0	0		
DoMYBR35	0.341813	0		
DoMYBR06	2.299533	13.10062667	5.697081393	up
DoMYBR16	0.03708	0.537155667		
DoMYBR18	9.407159	556.9604667	59.20602032	up
DoMYBR31	1.952981	31.13325	15.94140205	up
DoMYBR39	4.47755	49.17674667	10.98295787	up
DoMYBR40	8.42764	1.693619	0.200960047	down
DoMYBR10	15.27545	11.598706	0.759303719	
DoMYBR14	11.42285	20.17338667	1.76605598	up
DoMYBR19	15.01513	22.08029	1.470535726	
DoMYBR22	4.663029	5.574259	1.195415813	
DoMYBR25	24.50326	27.71872333	1.131225786	
DoMYBR30	1.736576	2.34203		
DoMYBR37	11.17866	10.71566	0.958581798	
DoMYBR13	22.04083	23.38211667	1.060854796	
DoMYBR28	4.975831	3.699522		
DoMYB3R1	6.781812	6.495187	0.957736174	
DoMYB3R2	0.82749	1.829341667		
DoMYB3R3	35.20298	33.1073	0.940468676	
DoMYB3R4	9.480676	3.785145333	0.39924845	down
DoMYB4R1	4.065327	3.445904667		
AtMYBCDC	34.51648	35.77649	1.036504692	

Supplementary Table 5 The relative expression results of eight clades (C1 (AtMYB5), C2 (S6), C8 (S21), C26 (S2), C27 (S3), C30 (S8), C33 (S13), C34 (AtMYB26/AtMYB67/AtMYB103) and C35 (AtMYB46/AtMYB83)) of R2R3-MYB genes in the roots, stems and leaves of *A. thaliana* and *P. equestris*.

Gene name	Root	Stem	Leaf
AtMYB103	9.966667	665.3333	9.413333
AtMYB105	6.323333	7.1	6.933333
AtMYB110	2.361667	5.96	8.09
AtMYB113	19.93333	17.1	36.2
AtMYB114	1.02	4.883667	1.865
AtMYB117	4.433333	8.943333	7.12
AtMYB13	58.33333	14.46667	19.1
AtMYB14	19.86667	2.718333	3.1
AtMYB15	518.6667	2.29	52.9
AtMYB20	16.16667	208.6667	35.46667
AtMYB26	1.202	2.846667	4.763333
AtMYB40	28.43333	3.074333	2.045333
AtMYB42	6.71	17.33333	0.609667
AtMYB43	83.16667	193.6667	7.486667
AtMYB46	28.76667	157.3333	27.7
AtMYB5	10.82333	9.843333	12.67333
AtMYB50	22.1	19.1	43.73333
AtMYB52	8.606667	185	0.737667
AtMYB54	14.5	29.13333	7.97
AtMYB55	26.4	155.3333	21.13333
AtMYB56	17.73333	21.6	7.886667
AtMYB58	13.9	104.2333	15.46667
AtMYB63	34.6	212	10.02333
AtMYB69	12.71333	134.6667	7.436667
AtMYB75	5.346667	6.37	14.59667
AtMYB67	68.73333	77.63333	2.533333
AtMYB83	4.076667	20.3	4.943333
AtMYB85	2.429	138	3.173
AtMYB86	68.73333	77.63333	2.533333
AtMYB90	1.131333	0.678	0.924333
AtMYB99	6.403333	1.74	1.506667
PeMYB01	0	2.854621	0
PeMYB04	0.28955	3.408825	0
PeMYB10	0	17.8708	0.582795
PeMYB101	0	0.199517	0
PeMYB104	1.124303	2.725103	0.463393
PeMYB108	2.182595	4.046139	2.878657
PeMYB11	9.222545	63.95267	22.87431
PeMYB111	27.7396	2.359148	8.986077

PeMYB14	11.46429	47.42832	0.657409
PeMYB16	16.7016	14.89145	0
PeMYB17	0.954557	0.480763	0
PeMYB19	0	0.069518	0
PeMYB20	5.499052	6.043446	42.96484
PeMYB26	0.438417	5.204778	0
PeMYB30	2.230082	3.589627	1.987357
PeMYB32	5.401922	0.173191	0
PeMYB36	3.934173	8.634538	0
PeMYB37	0	0.397707	0
PeMYB42	3.193897	5.461877	0.859689
PeMYB45	27.3777	18.73912	0
PeMYB47	0.55882	0	0
PeMYB48	9.626502	1.246978	0.91342
PeMYB54	0	0	0
PeMYB55	9.62684	11.33838	5.560722
PeMYB61	0	0	0
PeMYB62	2.351857	26.14073	0.387737
PeMYB65	1.806431	2.213286	0.305452
PeMYB67	5.853876	4.212898	0
PeMYB71	0	0	0.557159
PeMYB72	0	1.308305	0
PeMYB80	0	5.041933	4.45828
PeMYB81	0.691021	6.257898	1.051613
PeMYB82	0	2.21685	0.363968
PeMYB91	0	0.065201	0
PeMYB92	0	0.25579	0
PeMYB95	0.663831	1.241232	0

Supplementary Table 6 Relative expression levels of the 43 R2R3-MYB genes in the roots, stems and leaves of *D. officinale* by qRT-PCR.

Gene name	Root	Stem	Leaf
DoMYB01	1	0.121241	0.132222
DoMYB06	1	1.446962	0.383764
DoMYB42	1	0.987548	1.030883
DoMYB117	1	1.972139	0.953581
DoMYB74	1	4.192481	17.29642
DoMYB75	1	7.785834	30.47233
DoMYB86	1	1.135096	0.916702
DoMYB02	1	4.828104	1.803603
DoMYB111	1	0.516525	0.064562
DoMYB16	1	0.254134	0.236641
DoMYB26	1	1.186316	0.014551
DoMYB27	1	0.985686	0.143867
DoMYB31	1	6.953066	0.439771
DoMYB36	1	0.015741	0.070129
DoMYB45	1	0.28661	0.129912
DoMYB52	1	0.861736	0.852003
DoMYB64	1	1.025538	0.056001
DoMYB88	1	1.018411	0.017645
DoMYB97	1	0.507887	0.004986
DoMYB98	1	0.874075	0.219527
DoMYB10	1	1.025242	1.052421
DoMYB28	1	0.463426	0.240891
DoMYB54	1	0.032989	0.016111
DoMYB71	1	0.533067	0.361942
DoMYB78	1	3.981721	7.523926
DoMYB99	1	1.908522	1.175053
DoMYB46	1	0.533213	0.017349
DoMYB92	1	0.135686	0.047249
DoMYB07	1	1.975943	2.070756
DoMYB103	1	0.995242	0.032691
DoMYB67	1	0.252596	0.13382
DoMYB09	1	2.121944	0.071456
DoMYB110	1	1.133071	1.042858
DoMYB112	1	0.618706	1.762986
DoMYB115	1	1.983672	0.017051
DoMYB14	1	2.028543	0.018524
DoMYB29	1	0.918437	1.056521
DoMYB65	1	1.022214	1.003455
DoMYB81	1	0.879517	0.206898
DoMYB105	1	4.103133	0.019831
DoMYB63	1	1.002532	0.680111

DoMYB90	1	1.001697	0.002038
DoMYB17	1	3.755041	0.056756
